# Down-regulation of Skp2 expression inhibits invasion and lung metastasis in osteosarcoma

**DOI:** 10.1038/s41598-018-32428-9

**Published:** 2018-09-24

**Authors:** Yidan Zhang, Yoav S. Zvi, Brian Batko, Nikolas Zaphiros, Edmond F. O’Donnell, Jichuan Wang, Kenji Sato, Rui Yang, David S. Geller, Pratistha Koirala, Wendong Zhang, Xiuquan Du, Sajida Piperdi, Yang Liu, Deyou Zheng, Michael Roth, Jonathan Gill, Jinghang Zhang, Tingting Ren, Richard Gorlick, Xiaolin Zi, Bang H. Hoang

**Affiliations:** 1 Division of Pediatric Hematology, Oncology, Marrow & Blood Cell Transplantation, Children’s Hospital at Montefiore, Albert Einstein College of Medicine, Bronx, NY USA; 2 Department of Orthopedic Surgery, Montefiore Medical Center, Albert Einstein College of Medicine, Bronx, NY USA; 3 Department of Genetics, Albert Einstein College of Medicine, Bronx, NY USA; 4 Department of Neurology, Department of Genetics and Neuroscience, Albert Einstein College of Medicine, Bronx, NY USA; 5 Division of Pediatrics, University of Texas MD Anderson Cancer Center, Houston, TX USA; 6 Flow Cytometry Core, Albert Einstein College of Medicine, Bronx, NY USA; 7 Musculoskleletal Tumor Center, Beijing Key Laboratory for Musculoskeletal Tumors, Peking University People’s Hospital, Beijing, China; 8 Department of Urology, University of California, Irvine Medical Center, Orange, CA USA

## Abstract

Osteosarcoma (OS), the most common primary cancer of bone, exhibits a high propensity for local invasion and distant metastasis. This study sought to elucidate the role of S phase kinase-associated protein (Skp2) in osteosarcoma invasion and metastasis and to explore flavokawain A (FKA), a natural chalcone from kava extract, as a potential Skp2 targeting agent for preventing osteosarcoma progression. Skp2 was found to be overexpressed in multiple osteosarcoma cell lines, including 5 standard and 8 primary patient-derived cell lines. Patients whose tumors expressed high levels of Skp2 sustained a significantly worse metastasis-free (p = 0.0095) and overall survival (p = 0.0013) than those with low Skp2. Skp2 knockdown markedly reduced *in vitro* cellular invasion and *in vivo* lung metastasis in an orthotopic mouse model of osteosarcoma. Similar to Skp2 knockdown, treatment with FKA also reduced Skp2 expression in osteosarcoma cell lines and blocked the invasion of osteosarcoma cells *in vitro* and lung metastasis *in vivo*. Together, our findings suggest that Skp2 is a promising therapeutic target in osteosarcoma, and that FKA may be an effective Skp2-targeted therapy to reduce osteosarcoma metastasis.

## Introduction

Osteosarcoma is the most common primary malignant bone tumor that often occurs in children and young adults^1–3^. Multi-agent chemotherapy consisting of high-dose methotrexate, doxorubicin, and cisplatin (MAP) have led to significant improvements in survival from the 1960s to 1980s^4^. However, the survival rate for patients with metastatic or recurrent disease remains below 30%^5^, with no meaningful improvements over the last three decades^6^.

Targeted therapies for osteosarcoma are currently limited due to the genetic heterogeneity of these tumors^7^. Recently, several studies have sought to identify new potential therapeutic targets for osteosarcoma^5,8^. Skp2 is an F-box protein and E3 ubiquitin ligase that participates in many key cellular processes such as cell cycle regulation, senescence, apoptosis, and regulation of cancer stem cells^9–12^. Studies have shown that Skp2 is a substrate recognition component of the Skp1-Cullin1-F-box (SCF^Skp2^) complex, acting to ubiquitinate and degrade other proteins. Others have shown that Skp2 also promotes cellular invasion and metastasis by inducing RhoA and matrix metalloproteinases (MMPs)^13,14^. As such, Skp2 can interact with Myc, p300, and Miz1 in a complex to up-regulate RhoA transcription, independent of its SCF-E3 ligase activity^13^. Skp2 overexpression has been shown to be predictive of cancer progression and associated with a poor prognosis^15–17^. In prostate, gastric, and esophageal cancers, higher levels of Skp2 are associated with tumor metastasis and poorer survival, while down-regulation of Skp2 leads to inhibition of tumor growth and metastasis^12,14,16^. However, the involvement of Skp2 in the pathobiology of osteosarcoma remains unclear.

Kava (*Piper methysticum Forst*) is a plant that originates from the South Pacific Islands, often embedded in the regional culture as a ceremonial beverage. Flavokawains are major chalcones isolated from the kava root extract, acting as potent inducers of apoptosis and anti-proliferative agents in cancer, including bladder, breast, and prostate^18–20^. Previously, we have shown that flavokawain A (FKA) has an excellent safety profile by multiple short-term and long-term *in vivo* studies^21–23^. Recently, we reported that oral feeding with FKA prevents cancer progression in a transgenic model of prostate cancer by promoting Cullin-1 deneddylation, leading to degradation of Skp2^24^. Studies have shown that down-regulation of Skp2 leads to a blockade of G1/S or G2/M transition^25^. There are also reports that Skp2 plays a role in cancer metastasis^14,26,27^. Given our recent findings that FKA inhibits prostate cancer by degrading Skp2, we aimed to evaluate whether FKA has a therapeutic role in osteosarcoma by suppressing Skp2.

In this study, we sought to identify the functional role and prognostic significance of Skp2 in osteosarcoma. Secondly, we aimed to explore the potential role for FKA as a Skp2-targeted agent in preventing osteosarcoma progression. Our study revealed that high levels of Skp2 expression are predictive of a worse prognosis in osteosarcoma patients. Furthermore, we found that depletion of Skp2 by short hairpin RNA (shRNA) or by FKA results in down-regulation of Skp2 and several of its targets, leading to inhibition of invasion and metastasis in osteosarcoma.

## Results

### Skp2 is overexpressed in human osteosarcoma cells

**Figure 1:**
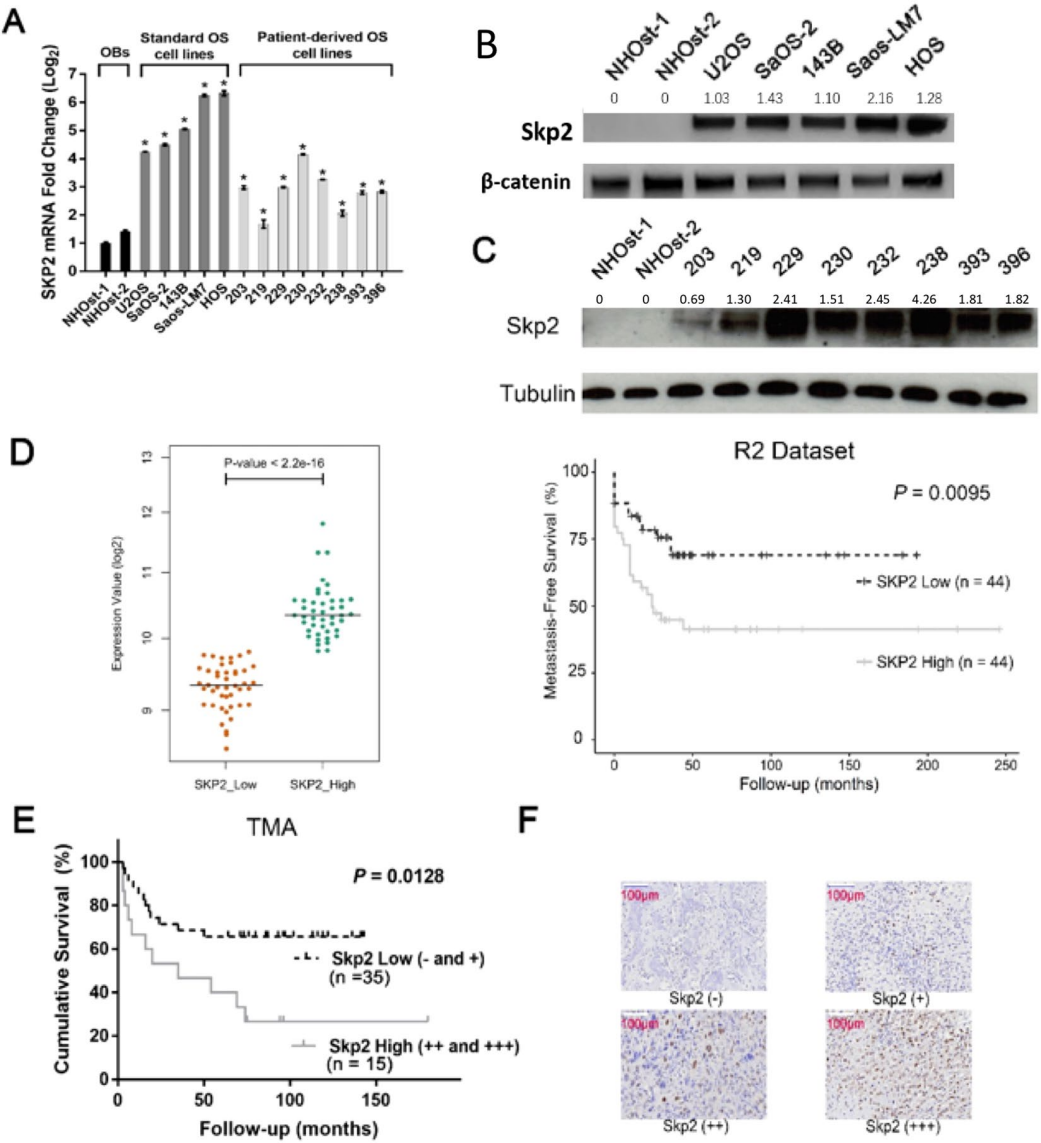
Skp2 is overexpressed in osteosarcoma cell lines and high Skp2 levels are correlated with a worse prognosis. (**A**) Quantitative RT-PCR. Skp2 mRNA expression in 5 standard and 8 patient-derived osteosarcoma cell lines was significantly increased compared to normal human osteoblasts (NHOst). **(B**,**C)** Skp2 protein levels were elevated in standard **(B)** and patient-derived **(C)** osteosarcoma cell lines compared to NHOsts. **(D)** Kaplan-Meier analysis. Raw Skp2 expression data was retrieved from NCBI GEO and correlated with survival data from the R2 platform. The median Skp2 mRNA expression was used as a cutoff to distinguish low vs. high expression. High Skp2 expression correlated significantly with a worse metastasis-free survival. **(E)** Tissue microarrays. Overall survival was compared in osteosarcoma patients whose tumors expressed low (- and +) vs. high (++ and +++) Skp2 (negative =<1% stained cells; (+) = 1–10%; (++) = 10–50%; (+++) =>50%). By log-rank test, the high Skp2 expression group sustained a worse overall survival than the low expression group. **(F)** Representative pictures of IHC scoring for Skp2. Statistical significance is indicated by: *p < 0.05, **p < 0.01, ***p < 0.001. Column: mean; Error bars: SD.

Skp2 mRNA levels were significantly elevated in several standard and patient-derived osteosarcoma cell lines compared to either normal human osteoblasts (NHOst-1) or human mesenchymal stem cell (MSC)-derived osteoblasts (NHOst-2) (p < 0.05) (Fig. 1A). Similarly, Skp2 overexpression in osteosarcoma cell lines was validated at the protein level using Western blot analysis (Fig. 1B,C, Supplementary Fig. 1). Since p27 has been reported as a substrate for Skp2-mediated ubiquitination, we also examined the expression of p27 in osteosarcoma cell lines^28^. Surprisingly, p27 protein levels are elevated in all osteosarcoma cell lines compared to NHOsts (Supplementary Fig. 1), suggesting an oncogenic role for this cell cycle regulator in osteosarcoma.

### High expression of Skp2 correlates with a worse survival in osteosarcoma patients

Metastasis-free survival was analyzed for 88 pre-treatment, high-grade osteosarcoma patients using data retrieved from NBCI GEO and the R2 platform. Two groups of patients were generated from the same cohort and the median Skp2 mRNA expression was determined and used as the cutoff to distinguish tumors with low versus high expression. Patients whose tumors expressed high Skp2 mRNA levels had a significantly worse metastasis-free survival compared to patients whose tumors expressed low Skp2 (p = 0.0095) (Fig. 1D), suggesting that Skp2 may have pro-metastatic activity in osteosarcoma.

To further evaluate the prognostic significance of Skp2 in osteosarcoma, we measured Skp2 expression by immunohistochemistry (IHC) using tissue microarrays (TMA) in which patient outcome data were available. Positive Skp2 immunostaining (graded from + to +++) was found in 36 of 50 (72%) samples. A total of 14 of 50 (28%) samples were found to be Skp2 negative (-). For survival analysis, the cohort was dichotomized into 2 groups: low (− to +) and high (++ to +++) Skp2 expression, based on the percentage of positive staining cells. Kaplan-Meier analysis and log-rank test revealed that overall survival of patients whose tumors expressed high Skp2 protein levels (n = 15) was significantly worse than for patients whose tumors expressed low Skp2 (n = 35) (p = 0.0128) (Fig. 1E), further demonstrating that Skp2 exerts oncogenic activity in osteosarcoma. Representative images of IHC grading for Skp2 are shown in Fig. 1F.

### Genetic knockdown of Skp2 reduces osteosarcoma proliferation and invasion

**Figure 2:**
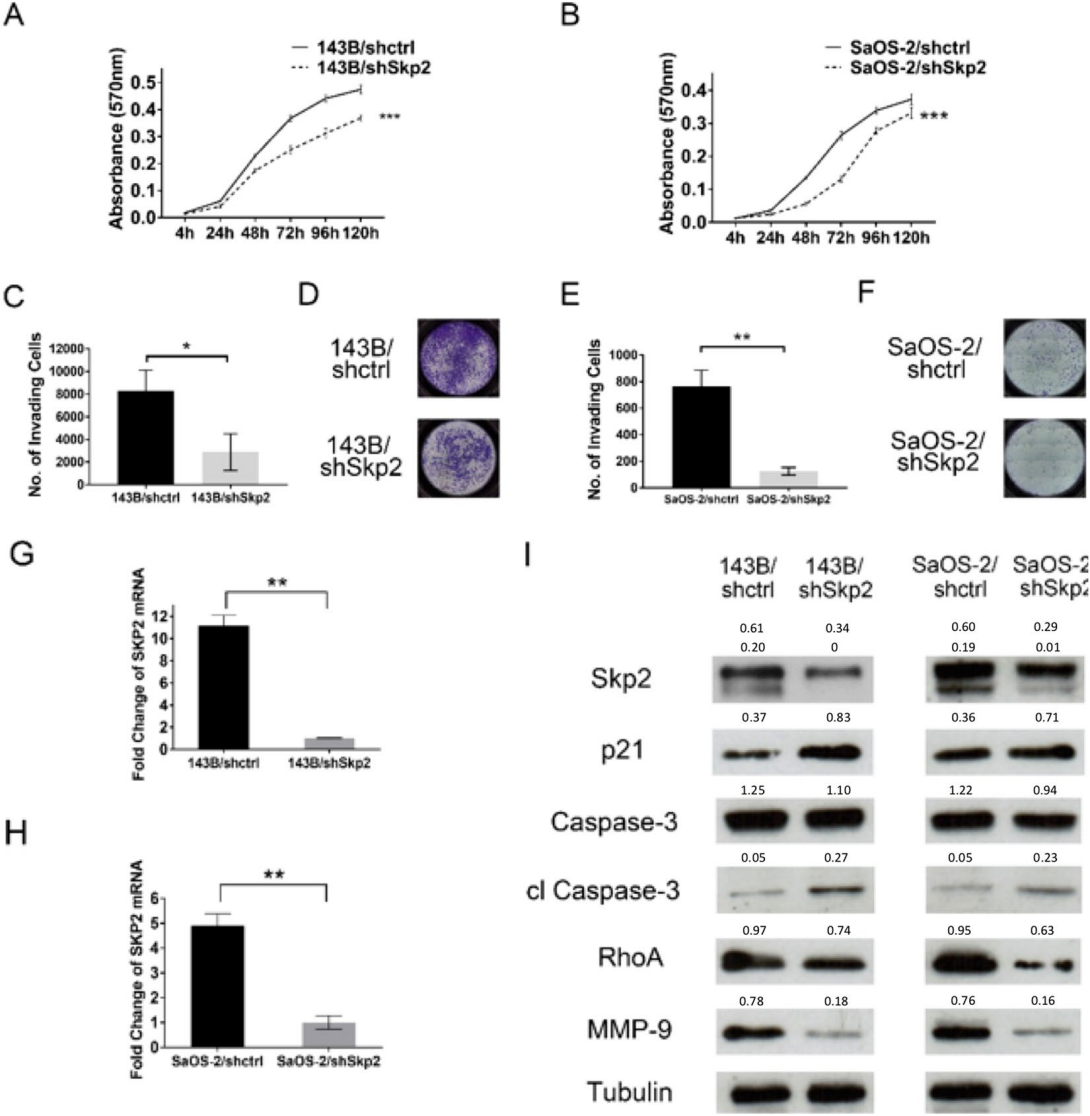
Proliferation, invasion, and mechanistic studies following knockdown of Skp2 in osteosarcoma cells. (**A**,**B**) MTT proliferation assays. Compared to control transfected cells, the proliferation of **(A)** 143B and **(B)** SaOS-2 Skp2-knockdown cells were significantly reduced. (**C**–**F**) Matrigel invasion assay. Compared to controls, invasion through Matrigel is significantly reduced in Skp2-knockdown 143B **(C)** and SaOS-2 **(E)** cell lines. **(D**,**F)** Representative pictures of the invasion chambers. Skp2 knockdown was confirmed by qRT-PCR in 143B (**G**) and SaOS-2 (**H**) cell lines. Western blots showed that p21 and cleaved caspase-3 levels were elevated, while RhoA and MMP-9 were reduced after Skp2 knockdown (**I**). Statistical significance is indicated by: *p < 0.05, **p < 0.01, ***p < 0.001. Column: mean; Error bars: SD.

Since patient survival data suggested that Skp2 may be a proto-oncogene in osteosarcoma, we next performed knockdown of Skp2 by shRNA to determine its biological effects on osteosarcoma cells. In MTT assays, Skp2 knockdown significantly reduced the proliferation of 143B and SaOS-2 cell lines (p < 0.001) (Fig. 2A,B). After Skp2 knockdown, Matrigel invasion assays showed 65.2% (2887 ± 1613 vs. 8307 ± 1804, p = 0.018) and 83.6% (123 ± 28 vs. 762 ± 125, p = 0.001) reductions in the migration of 143B and SaOS-2 cell lines, respectively (Fig. 2C–F). The efficiency of Skp2 knockdown was confirmed by qRT-PCR and Western blotting (Fig. 2G–I). Fig. 2I shows the two splice variants of Skp2 (46 and 48 kDa) as previously described^24^. In addition, protein levels of cell cycle regulator p21, along with cleaved caspase-3, were elevated following Skp2 knockdown (Fig. 2I), suggesting that Skp2 depletion likely leads to cell cycle arrest and apoptosis. Notably, both RhoA and MMP-9, which are positively regulated by Skp2 and play a role in osteosarcoma invasion^29,30^, were suppressed following Skp2 knockdown (Fig. 2I). However, expression of p27, an Skp2 substrate, was not significantly affected by Skp2 knockdown (Supplementary Fig. 2), suggesting that in osteosarcoma, p27 levels may be regulated by Skp2-independent mechanisms.

To demonstrate the specificity of Skp2 knockdown in 143B and SaOS-2, two additional unique shRNAs were used to target Skp2. As shown in Supplementary Fig. 3, knockdown of Skp2 using another shSkp2 construct also led to an increase in p21 and a decrease in RhoA protein levels. We found that all three shRNA constructs had similar effects on Skp2 target genes. Collectively, these results suggested that Skp2 has anti-apoptotic and pro-invasive activity in osteosarcoma cells.

### Genetic knockdown of Skp2 reduces osteosarcoma growth and metastasis *in vivo*

**Figure 3:**
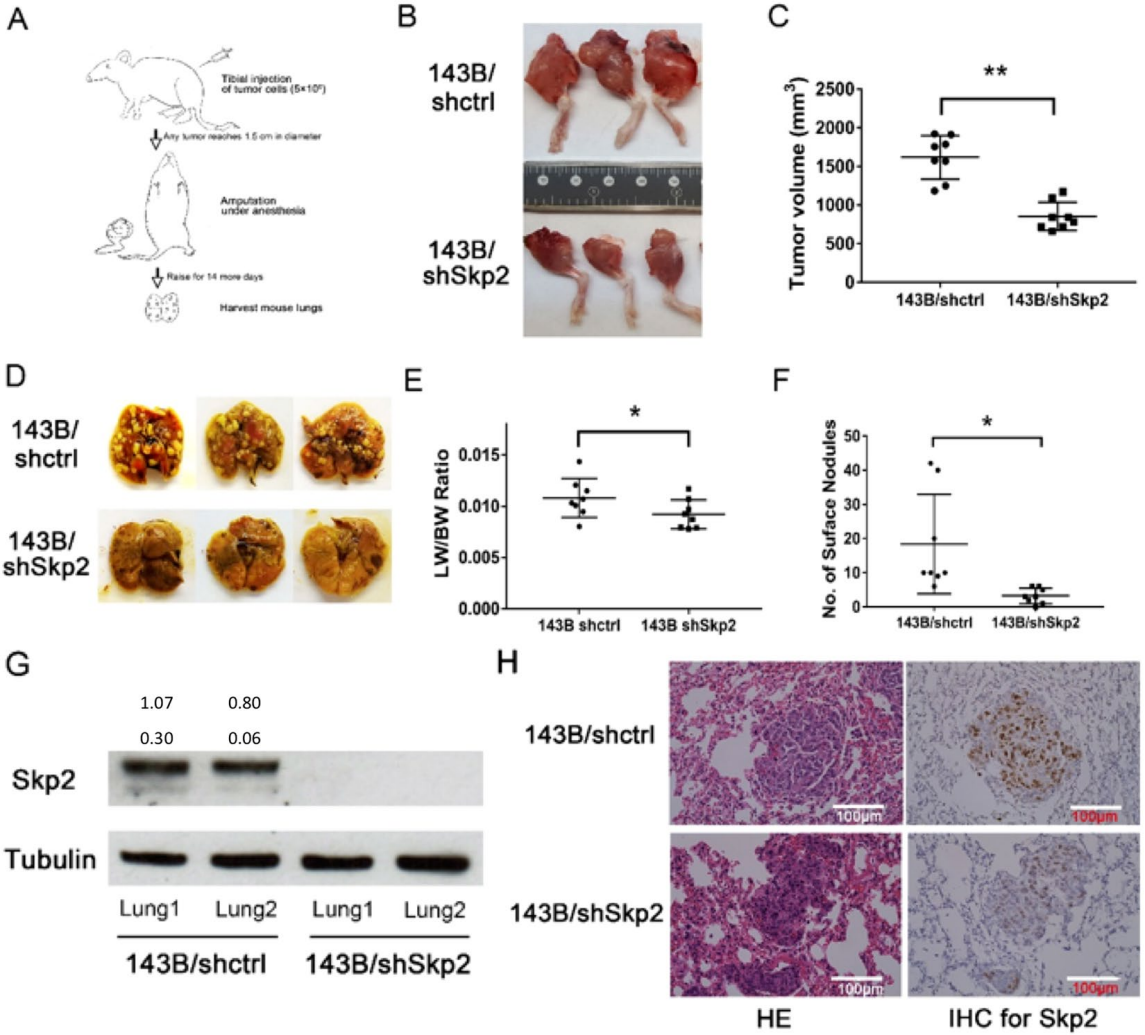
Skp2 knockdown inhibits tumor growth and lung metastasis in an orthotopic osteosarcoma model. (**A)** Experimental design. 5 × 10^5^ 143B/shctrl or 143B/shSkp2 cells were implanted into the proximal tibias of mice. Legs were amputated once any tumor reached 1.5 cm in diameter. Animals were kept for 2 weeks after amputation and then sacrificed. **(B)** Representative pictures of tibia tumors. **(C)** Tumor volumes of the Skp2-knockdown group were significantly than the control group. **(D)** Representative pictures of lungs harvested from mice injected with transfected 143B/shctrl and 143B/shSkp2 cell lines. **(E)** The mean lung weight (LW) normalized to body weight (BW) was significantly greater in the control group compared to the Skp2-knockdown group. **(F)** Skp2 knockdown significantly reduced the number of metastatic lung nodules. **(G)** Expression of Skp2 from two different lungs was examined by Western blot. No Skp2 expression was observed in143B/shSkp2 lung tissue. **(H)** Representative pictures of hematoxylin-eosin (HE) and IHC staining of lung tissues. Lower levels of Skp2 immunostaining were detected in metastatic lung nodules from the 143B/shSkp2 group. Statistical significance is indicated by: *p < 0.05, **p < 0.01, ***p < 0.001. Column: mean; Error bars: SD.

To evaluate the impact of Skp2 knockdown on osteosarcoma growth and lung metastasis, we used an orthotopic mouse model in which tumor cells were injected into the proximal tibia of SCID mice (Fig. 3A). After 143B/shctrl and 143B/shSkp2 cells were injected, tumor-bearing legs were amputated in all animals when any tumor reached 1.5 cm in diameter. We found that the mean tumor volume in the143B/shSkp2 group (852 ± 184 mm^3^) was significantly lower than that of the 143B/shctrl group (1618 ± 281 mm^3^) (p < 0.01) (Fig. 3B,C).

After tumor-bearing legs were amputated, the animals were maintained for two additional weeks before being euthanized and their lungs were harvested. The mean lung weight, normalized to body weight, of the 143B/shSkp2 group (0.92 ± 0.14) was significantly less than that of the control group (1.08 ± 0.19) (p < 0.05) (Fig. 3E). In addition, a significantly higher number of surface lung nodules were observed in the control group (p < 0.05) (Fig. 3D,F). Western blot analysis and IHC staining showed higher levels of Skp2 expression in the lung tissues of the control group compared to the Skp2-knockdown group (Fig. 3G,H). Using IHC, we found that Skp2 knockdown did not significantly affect the levels of p27 in the metastatic lung nodules (Supplementary Fig. 4).

### FKA inhibits osteosarcoma cellular proliferation and invasion

**Figure 4:**
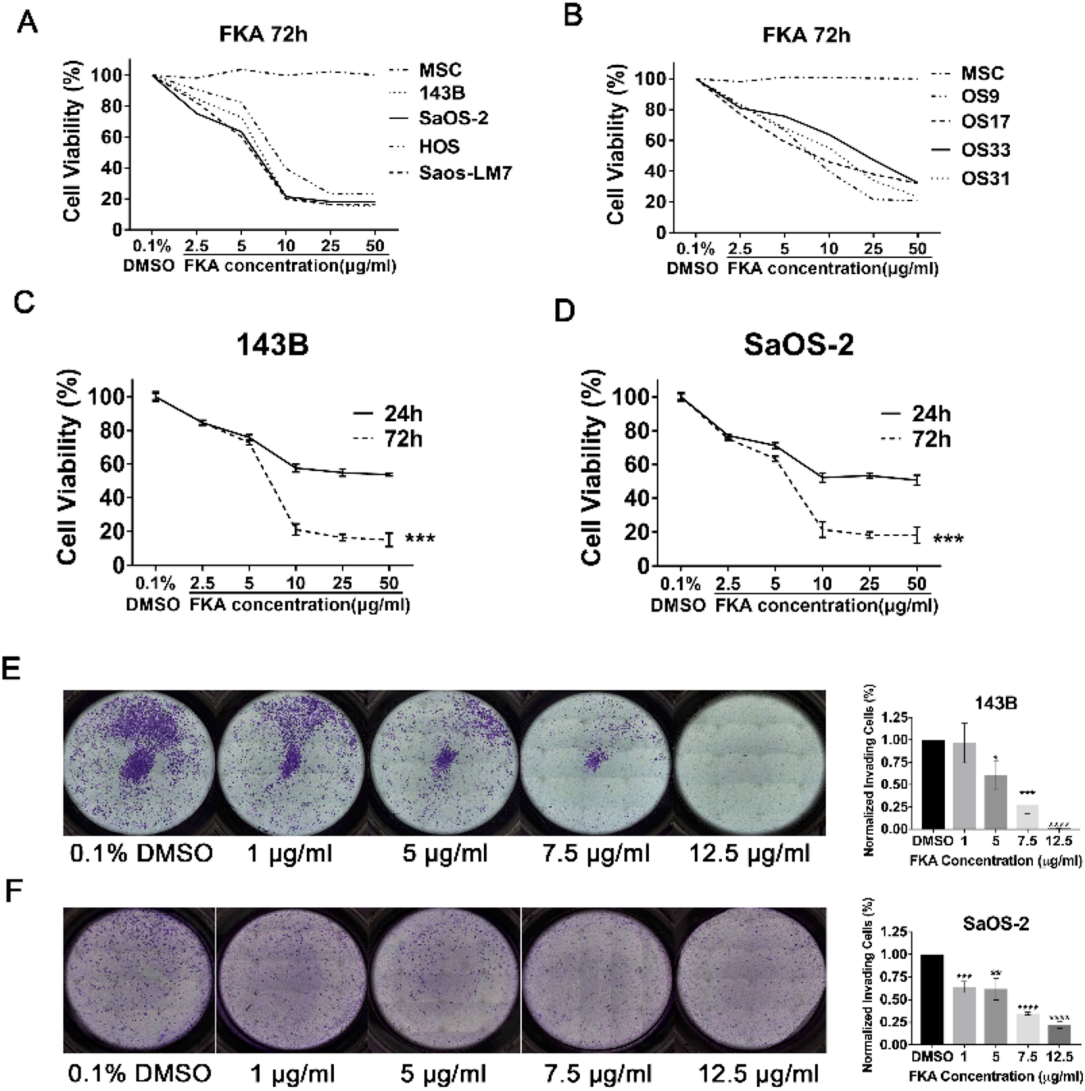
FKA inhibits the growth and invasion of osteosarcoma cells. (**A**,**B**) Standard osteosarcoma cell lines (143B, SaOS-2, HOS, Saos-LM7) and patient-derived xenograft cell lines (OS9, OS17, OS31, and OS33) were treated with serial dilutions of FKA for 72 hours and cell viability was determined using MTT assays. Representative curves from 3 independent experiments are shown. Human mesenchymal stem cells (MSC) were used as a normal control. Compared to treatment for 24 hours, 72 hours of FKA treatment resulted in significantly reduced cell viability in 143B **(C)** and SaOS-2 **(D)** cell lines. Two-way ANOVA was used for statistical analysis. **(E**,**F)** Matrigel invasion assays. FKA treatment of 143B **(E)** and SaOS-2 **(F)** reduced the percent of invading cells in a dose-dependent manner, normalized to the corresponding MTT assays. Statistical significance is indicated by: *p < 0.05, **p < 0.01, ***p < 0.001, ****p < 0.0001. Column: mean; Error bars: SD.

We next treated osteosarcoma cell lines with FKA to examine its effects on cellular growth and invasion. MTT assays were performed on standard osteosarcoma cell lines (143B, SaOS-2, HOS, and Saos-LM7) and patient-derived xenograft (PDX) osteosarcoma cell lines (OS9, OS17, OS33, and OS31) treated with serial dilutions of FKA. MSCs were used as a normal control. After 72 hours, FKA treatment resulted in a significant decrease in cell viability in all standard osteosarcoma cell lines. At 10 μg/ml, FKA inhibited the growth of 143B, SaOS-2, and Saos-LM7 cells by approximately 80%. The half-maximal inhibitory concentration (IC_50_) of FKA at 72 hours was approximately 7.5 μg/ml (Fig. 4A). Additionally, the inhibitory effect of FKA was observed in four PDX cell lines (Fig. 4B), while no significant cytotoxicity was detected in MSCs. Furthermore, a comparison of 1 day vs. 3 days of treatment showed that prolonged exposure to FKA led to a significant dose-dependent decrease in cell viability (p < 0.001) (Fig. 4C,D). To account for the effects of FKA on cell viability in invasion assays, Matrigel assays were performed on 143B and SaOS-2 and the percent of invading cells were normalized to cell viability for each FKA dose, using the corresponding MTT assay.

Matrigel invasion assays at 24 hours showed that FKA significantly inhibited 143B and SaOS-2 cellular invasion in a dose-dependent manner. Compared to controls, a 5 μg/ml dose of FKA in 143B and SaOS-2 resulted in 39.5% (p = 0.0138) and 38.3% (p = 0.0053) reduction in invading cells, respectively. Likewise, treatment with 7.5 μg/ml of FKA reduced cellular invasion by 73% (p = 0.0002) and 65.4% (p < 0.0001), while 12.5 μg/ml of FKA reduced cellular invasion by 98.5% (p < 0.0001) and 78.1% (p < 0.0001) in 143B and SaOS-2 cells, respectively (Fig. 4E,F).

### FKA decreases Skp2 expression in osteosarcoma cell lines

**Figure 5:**
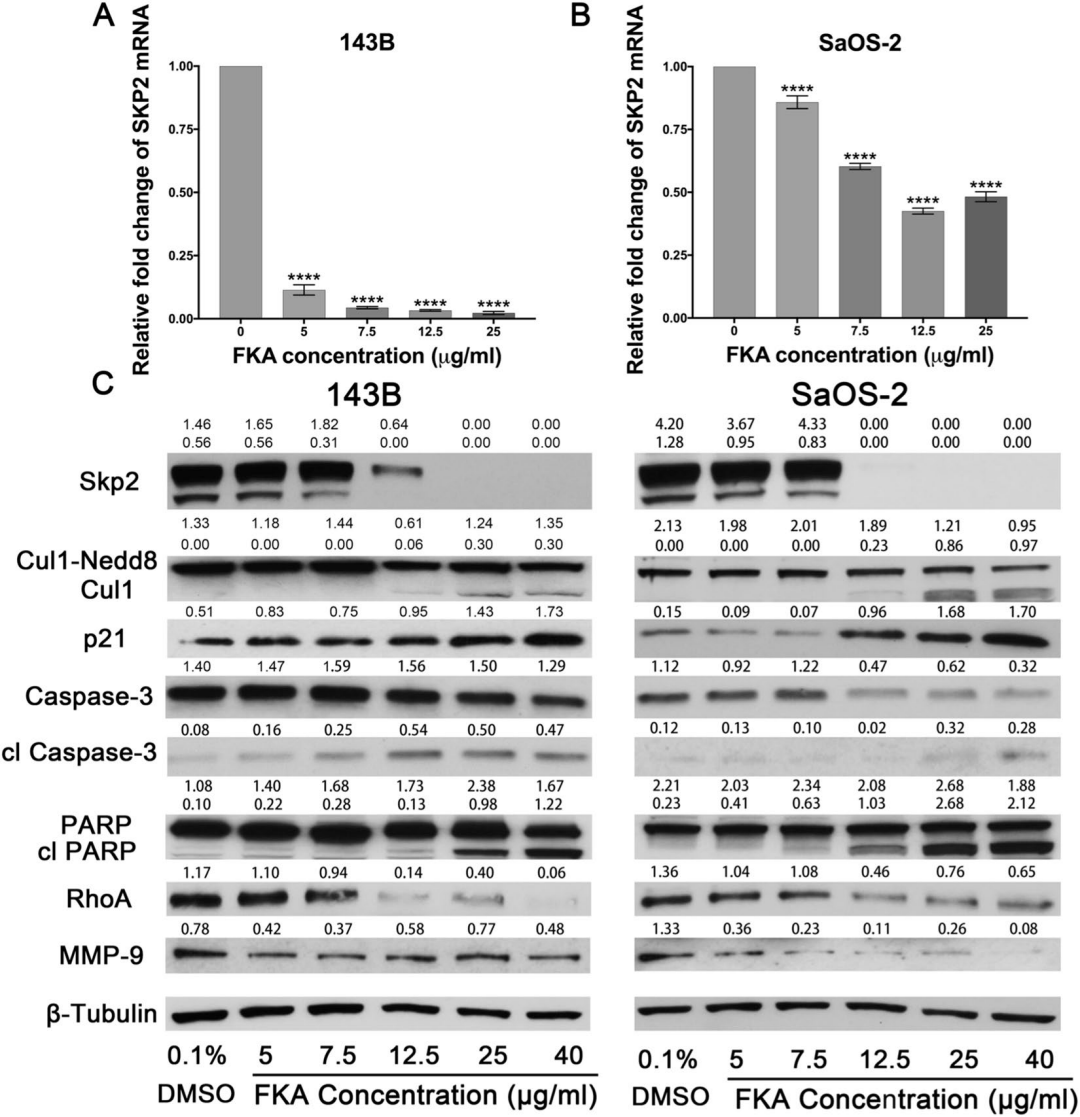
FKA exerts inhibitory effects by targeting Skp2 in osteosarcoma cells. Quantitative RT-PCR. Treatment with FKA markedly decreased SKP2 mRNA levels in 143B **(A)** and SaOS-2 **(B)** cell lines. **(C)** Western blot showing FKA-mediated suppression of Skp2 and Cul1-Nedd8 in a dose-dependent manner. Levels of the cell cycle regulator p21 (a known Skp2 target) were also elevated, along with the apoptosis markers cleaved Caspase-3 and cleaved PARP. Other Skp2 targets such as RhoA and MMP-9, were also inhibited by FKA. Independent t-tests were used to evaluate differences in qRT-PCR data. Statistical significance is indicated by: ****p < 0.0001. Column: mean value; Error bars: SD.

To determine the mechanisms underlying FKA-induced inhibition of Skp2, we next treated osteosarcoma cell lines 143B and SaOS-2 with increasing concentrations of FKA and examined its effects on Skp2. First, we found a negative correlation between Skp2 mRNA levels and FKA concentration by qRT-PCR. At 12.5 μg/ml of FKA, Skp2 mRNA levels were reduced by more than 90% (p < 0.0001) in 143B cells (Fig. 5A) and by 58.5% (p < 0.0001) in SaOS-2 cells (Fig. 5B). Similarly, Western blot analysis showed that Skp2 expression was suppressed by FKA in a dose-dependent manner. Given our previous data showing that FKA induces Skp2 degradation via deneddylation of Cullin1^24^, we next examined the expression of Cul1-Nedd8 by Western blot analysis. Our data revealed that neddylation of Cullin1, which prevents Skp2 degradation, was inhibited by FKA in 143B and SaOS-2 cell lines (Fig. 5C). Taken together, these data suggest that FKA may reduce Skp2 expression in osteosarcoma through both transcriptional and post-translational mechanisms.

Similar to the effects of Skp2 knockdown (Fig. 2I), FKA also induced an accumulation of p21 and cleaved caspase-3 in osteosarcoma cell lines (Fig. 5C). Conversely, overexpression of Skp2 in osteosarcoma cell lines treated with the same FKA doses abrogated the effects of FKA on p21 and cleaved PARP (Supplementary Fig. 5). To determine whether Skp2 down-regulation is a cause or consequence of apoptosis due to FKA, we performed a time-course experiment using 143B and SaOS-2 cell lines treated with 12.5 µg/ml of FKA (dosage with the most significant effects in both cell lines). Western blot analysis demonstrated that FKA suppresses Skp2 at 6 hours, while no significant apoptosis by PARP cleavage was observed until 12 to 24 hours (Supplementary Fig. 6). These findings suggested that FKA-mediated suppression of Skp2 is more likely to be the cause than the consequence of apoptosis.

FACS analysis showed that FKA induces G2/M phase cell cycle arrest, and that apoptosis becomes more evident at FKA concentrations approaching 25 μg/ml (Supplementary Fig. 7). In addition to caspase-3, we also observed an increase in cleaved PARP, suggesting that FKA is effective in inducing apoptosis of osteosarcoma cells (Fig. 5C). Interestingly, the expression of both RhoA and MMP-9 was reduced by relatively low doses of FKA (5–12.5 μg/ml) (Fig. 5C), consistent with our observation that cellular invasion was suppressed at similar doses (Fig. 4E,F). To further characterize MMP-9 gelatinase activity in response to increasing concentrations of FKA, we performed zymogram assays. As shown in Supplementary Fig. 8, FKA treatment led to a decrease in the gelatinolytic activity of MMP-9 in SaOS-2 cell line at higher doses. However, FKA treatment did not consistently reduce MMP-9 activity in 143B cells, suggesting that other non-gelatinase functions of MMP-9 may be affected by FKA^31^. Furthermore, we examined the Skp2 substrate p27 and found that FKA treatment is associated with a decline in p27 protein levels, concomitant with decreased invasion in both 143B and SaOS-2 cell lines (Supplementary Fig. 9, Fig. 4E,F).

### Oral treatment with FKA inhibits osteosarcoma lung metastasis *in vivo*

**Figure 6:**
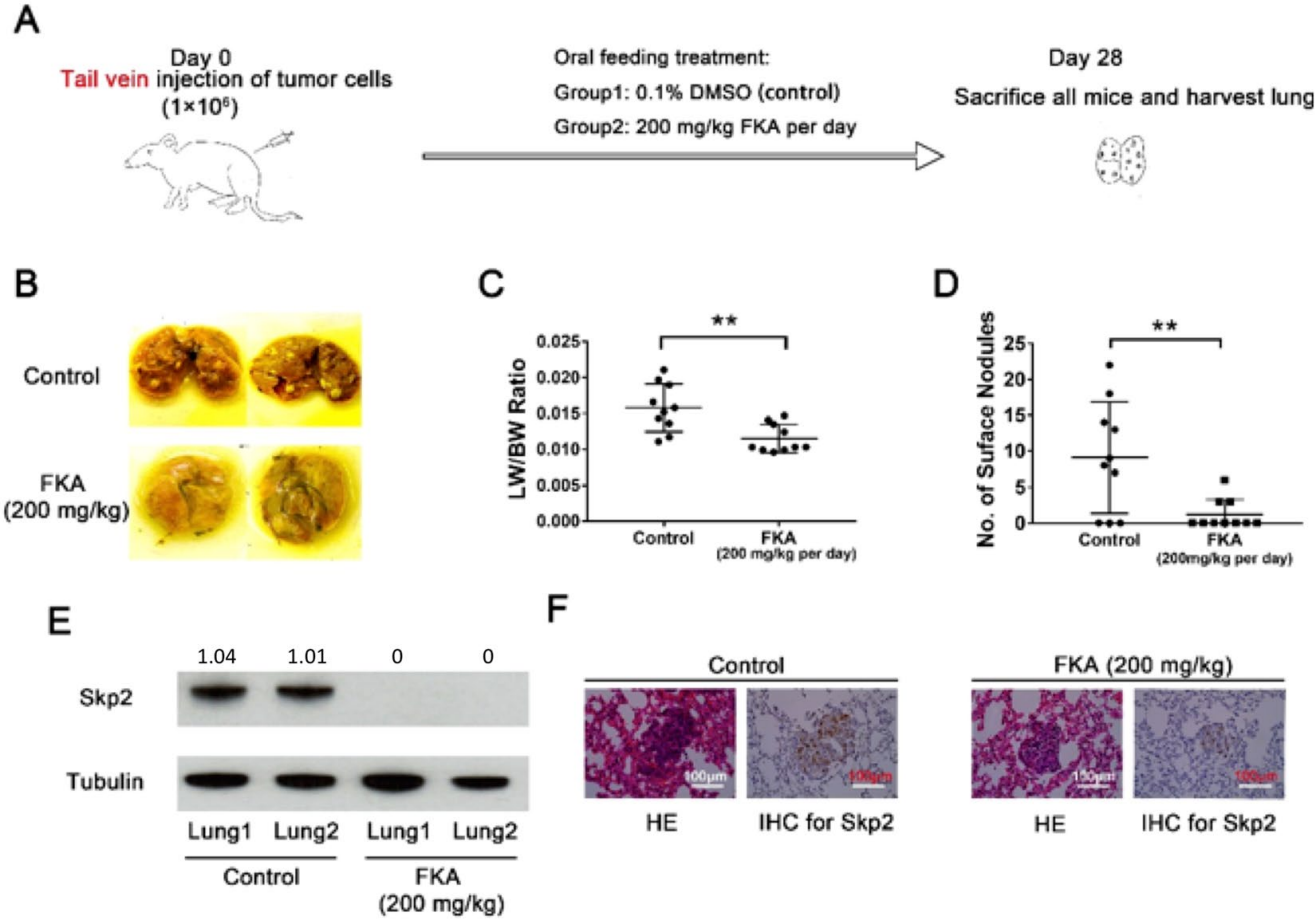
Oral treatment with FKA blocks osteosarcoma lung metastasis *in vivo*. (**A**) Experimental design. **(B**,**D)** FKA treatment significantly reduced the number of metastatic surface lung nodules. **(C)** Mean normalized lung weight (LW) to body weight (BW) was significantly lower in the FKA-treated group compared to the FKA-treated group. **(E)** Western blot showing Skp2 protein expression in two representative lung specimens harvested from control and FKA-treated mice. Skp2 expression in lung tissue lysates was markedly suppressed after FKA treatment **(F)** Representative pictures of HE and IHC staining of lung tissues. Independent t-tests were used to compare differences in the LW/BW ratio and lung nodules between control and FKA-treated groups. Statistical significance is indicated by: *p < 0.05, **p < 0.01, ***p < 0.001. Column: mean; Error bars: SD.

To assess the *in vivo* anti-metastatic effects of FKA, 200 mg/kg were administered daily by oral gavage, three days after injection of 143B cells into the tail vein of SCID mice (Fig. 6A). Compared to the vehicle control group, significantly fewer metastatic pulmonary nodules were observed in the FKA treated group (p < 0.01) (Fig. 6B,D). Additionally, the normalized lung weights at the endpoint were significantly lower in the FKA treated group (p < 0.01) (Fig. 6C,D). Western blot analysis demonstrated that Skp2 levels were higher in lung tissue of the control group compared to those of the FKA-treated group (Fig. 6E), which was confirmed by IHC staining (Fig. 6F). In addition, immunostaining also demonstrated decreased levels of p27 in metastatic lung nodules from the FKA-treated group (Supplementary Fig. 10).

Using the orthotopic tibia injection model, we found increased apoptosis of the primary tumor by TUNEL after a high dose of FKA (600 mg/kg), compared to vehicle control. However, no significant difference in tumor volume was observed between FKA-treated and control groups (Supplementary Fig. 11).

## Discussion

Osteosarcoma is a highly malignant bone tumor in adolescents and young adults that often metastasizes. In this report, we have identified the F-box protein Skp2 as a proto-oncogene that plays a role in osteosarcoma progression and metastasis. We showed that Skp2 is significantly overexpressed in osteosarcoma cell lines and that high levels of Skp2 are predictive of a worse metastasis-free and overall survival in patients (Fig. 1). In addition, our data showed that both genetic knockdown of Skp2 and pharmacologic suppression of Skp2 by FKA inhibits osteosarcoma invasion and lung metastasis (Figs 2–6). Additionally, our study demonstrated that FKA inhibits the growth of osteosarcoma cell lines by inducing G2/M cell cycle arrest and apoptosis (Supplementary Fig. 7).

The development of targeted therapies for osteosarcoma has been stagnant for decades due to a large amount of heterogeneity that are inherent in these tumors^32^. There is no effective treatment for patients with widely metastatic disease or those who respond poorly to systemic chemotherapy^5^. Furthermore, conventional chemotherapy for osteosarcoma is limited by significant, often dose-limiting, side effects. Therefore, there is an urgent need to develop novel therapeutic agents that target underlying mechanisms of invasion and metastasis in osteosarcoma. In this study, we provide evidence that Skp2 is a therapeutic target that plays an important role in the invasion and metastasis of osteosarcoma. We also demonstrated that Skp2 can be inhibited effectively by FKA (Fig. 5), a natural chalcone from kava that shows minimal toxicity in normal tissues and organs^21,23,24^.

Skp2 is an essential component of the Myc/Skp2/Miz1 complex, which induces RhoA gene transcription^13^. In osteosarcoma, our observation that RhoA is suppressed after depleting Skp2 is consistent with previous reports. Since the RhoA GTPase plays a crucial role in the invasiveness and metastasis of multiple cancers^29,33–37^, it is likely that FKA or Skp2 knockdown blocks RhoA, leading to inhibition of invasiveness and lung metastasis in osteosarcoma. Furthermore, previous studies have shown that Skp2 promotes the expression of matrix metalloproteinase (MMP)^14^, thus enhancing metastatic potential^38^. In the current study, we demonstrated that both FKA and Skp2 knockdown are effective in suppressing MMP-9, suggesting a common anti-invasive mechanism for both strategies. Together, our results suggest that FKA exerts its anti-metastatic effects in osteosarcoma by suppressing Skp2.

FKA has been shown to exert inhibitory effects on a variety of cancer cells^19,39^. Here, we demonstrated that FKA inhibits the proliferation of multiple standard and patient-derived osteosarcoma cell lines (Fig. 4). However, this reduction in proliferative activity by FKA may be partially attributed to an increase in apoptosis. Additionally, we demonstrated that FKA effectively inhibits osteosarcoma invasion *in vitro* and lung metastasis *in vivo* (Figs 4 and 6). Our data suggested that FKA may deplete Skp2 in osteosarcoma cells, through deneddylation and protein degradation, similar to what has been reported in prostate cancer^24^. However, unlike prostate cancer, treatment with FKA also leads to down-regulation of Skp2 mRNA, suggesting that FKA functions at a transcriptional level in osteosarcoma (Fig. 5). Furthermore, treatment with FKA negatively influences the expression of RhoA and MMP-9, along with decreasing cellular invasiveness. This result is consistent with our findings that FKA exerts an anti-metastatic efficacy *in vivo* (Fig. 5). Taken together, we surmise that the anti-invasive and anti-metastatic effects of FKA in osteosarcoma cells are the result of FKA-induced depletion of Skp2.

Although p27 is a known substrate for Skp2-mediated ubiquitination in many cancers, our results demonstrated that levels of p27 were not significantly affected by Skp2 knockdown in osteosarcoma cell lines. A similar lack of correlation between Skp2 and p27 levels has been reported in diffuse large B-cell lymphoma, suggesting that other factors may contribute to deregulation of p27 in some cancers^40^. Interestingly, treating osteosarcoma cell lines with FKA also led to a decline in both Skp2 and p27 protein levels, along with decreased invasion and metastasis. Our data is consistent with recent findings by Li *et al*. demonstrating that knockdown of p27 in osteosarcoma cell lines leads to reduced motility and invasion^41^. These authors found that cytoplasmic accumulation of p27 in osteosarcoma cells is associated with enhanced metastatic potential and a worse prognosis, suggesting that p27 is an oncogene with pro-metastatic functions in osteosarcoma. Collectively, our results indicate that FKA may affect p27 levels via Skp2-independent mechanisms in osteosarcoma.

Although FKA has a significant anti-metastatic efficacy, we did not observe a reduction in overall tumor volume in our *in vivo* models (Supplementary Fig. 11). Further, our *in vitro* experiments suggested that FKA has anti-invasive effects even at low concentrations, but induces apoptosis and cell cycle arrest at relatively higher concentrations (Figs 4 and 5). Therefore, it is plausible that a higher serum concentration of FKA may be needed to achieve local tumor control in our experimental models. Another possible scenario is that high-dose FKA may induce more tumor necrosis without affecting local tumor size. Consistent with this possibility, we observed an increase in apoptosis in primary tumors at FKA doses of 600 mg/kg (Supplementary Fig. 11). Nonetheless, the low-toxicity profile of FKA may allow us to use it concurrently with conventional chemotherapy to reduce both local tumor burden as well as lung metastasis. In the future, more comprehensive studies will be needed to determine whether FKA has synergistic effects in combination with standard chemotherapy drugs for osteosarcoma.

During the course of our investigation, Ding and colleagues reported that overexpressing Skp2 leads to enhanced cellular growth, motility, and invasion in osteosarcoma, while increased apoptosis was observed in cell lines depleted of Skp2^42,43^. Although these authors did not analyze Skp2 in an *in vivo* model, their findings certainly corroborate our hypothesis that Skp2 plays an important oncogenic role in osteosarcoma pathobiology. To our knowledge, there is only one prior single-institutional study using immunohistochemistry to correlate Skp2 protein expression with osteosarcoma relapse and metastasis^44^. In our study, a multi-pronged approach was undertaken to provide evidence that Skp2 is an important prognostic marker in osteosarcoma. First, we used multiple patient-derived and established cell lines to demonstrate that Skp2 mRNA and protein are overexpressed in osteosarcoma compared to normal osteoblasts. Second, our analysis of the public Gene Expression Omnibus (GEO) dataset provided robust evidence that Skp2 mRNA expression is negatively correlated with metastasis-free survival. Finally, to compliment our mRNA expression data, we also performed immunohistochemistry of tissue microarrays to demonstrate that high Skp2 expression significantly correlates with a worse overall survival. Together, these results establish the prognostic significance of Skp2 in osteosarcoma in a much more rigorous fashion than previously reported.

In summary, our study identified the F-box protein Skp2 as a potential therapeutic target for osteosarcoma. Our findings revealed that Skp2 is a prognostic marker that is frequently overexpressed in osteosarcoma, and that genetic knockdown of Skp2 effectively inhibits osteosarcoma invasion and metastasis. Our results suggest that the anti-tumor efficacy of FKA is related to its ability to suppress Skp2. FKA clearly demonstrates a capacity to suppress *in vitro* invasion and *in vivo* lung metastasis, often the main culprit for treatment failure in osteosarcoma. Although FKA also induced cell cycle arrest and apoptosis in osteosarcoma cells, and these effects were more pronounced at higher doses. The utility of FKA, however, may lie in its potential to reduce osteosarcoma progression by inhibiting lung metastasis. As such, our study provides a framework for further investigation of the clinical utility of flavokawain A in preventing the progression of osteosarcoma as well as other Skp2-dependent cancers.

## Materials and Methods

### Cell culture and preparation of FKA

Standard human osteosarcoma cell lines 143B, SaOS-2, HOS, and U2OS (ATCC); the metastatic cell line, SaOS-LM7 (gift from Dr. Eugenie Kleinerman, MD Anderson Cancer Center, Houston, TX); eight patient-derived osteosarcoma cell line (203, 219, 229, 230, 232, 238, 393, 396)^45^; and patient-derived xenograft lines, OS9, OS17, OS31, and OS33 (Pediatric Preclinical Testing Program)^46^ were maintained in EMEM supplemented with 10% FBS. The human osteoblast lines NHOst-1 (Lonza CC-2538) and NHOst-2 (ATCC PCS-500-012) were obtained by incubation in osteogenic differentiation media (PT-3002, Lonza, Breda, Netherlands) for 21 days. All cells were cultured at 37 °C in a humidified incubator with 5% CO_2_.

Pure FKA (F4502) was obtained from LKT LABS (St Paul, MN, USA), dissolved in dimethyl sulfoxide (DMSO), aliquoted at concentrations of 25 mg/ml, and stored at −20 °C for use.

### Cell proliferation assay

The MTT assay [(1-(4,5-Dimethylthiazol-2-yl)−3,5-diphenylformazan)] (Sigma-Aldrich Co. LLC, MO, USA) was used to determine cell proliferation and viability as previously described^47^. For proliferation assays, cells were plated into 96-well plates at a density of 1.5 × 10^3^ cells in 100 μl of growth medium. Cell proliferation was compared between osteosarcoma cell lines transfected with vehicle control and Skp2-knockdown plasmids. For viability assays, cells were cultured in 96-well plates at a density of 3 × 10^3^ cells in 100 μl of growth medium overnight. Following treatment with increasing doses of FKA or 0.1% DMSO as a control for 24 or 72 hours, MTT assay was conducted for cell viability measurement. Dose-response curves were created using Prism software to measure viability relative to vehicle-treated cells (GraphPad, La Jolla, CA, USA).

### Invasion assay

Cell invasion was evaluated using the 24-well BioCoat Matrigel invasion chamber (354480, Corning, NY, USA) as previously described^47^. The effect of FKA on cell invasion was tested using concentrations below the IC_50_ of the anti-proliferative effect of FKA, as predetermined by cell viability assays. The percent of invading cells were normalized to cell viability for each FKA dose using the corresponding MTT assays. Normalized invading cells (%) = (# of invaded cells)/(initial no. of seeded cells) * (% viability at corresponding FKA dose).

### Quantitative RT-PCR

Total RNA was isolated using the RNAeasy Mini Kit (Qiagen, Hilden, Germany) according to the manufacturer’s instructions. Reverse transcription of total RNA was performed using the SuperScript III one-step RT-PCR system (Invitrogen, Cambridge, MA, USA). Real-time PCR was performed as previously described^48^. Relative fold changes of mRNA expression compared to controls were calculated using the comparative Ct method utilizing Thermo Fisher Cloud. Prism software was used to analyze results.

### Western blot analysis

For Western blots, 20ug of protein lysate was separated electrophoretically on denaturing SDS-polyacrylamide gel, transferred to nitrocellulose membranes, blocked with 5% TBSTM, incubated with primary antibodies and appropriate secondary antibodies followed by detection with ECL reagents as previously described^49^. Protein bands were visualized by autoradiography. Image Studio 5.2 software was used for densitometric quantification of protein bands, adjusted to tubulin loading controls. The intensity of each tested marker is presented as a ratio of a tested mark/tubulin. Anti-Skp2 antibody was obtained from Invitrogen (32–3300, Thermo Fisher Scientific, Cambridge, MA, USA). Two splicing variants of Skp2 were detected according to the antibody manufacturer. Antibodies against p21 (2947), p27^kip1^(3686), RhoA (2117), MMP-9 (13667), PARP (9542 s), and tubulin (2146) were purchased from Cell Signaling Technology (Beverly, MA, USA). Antibodies against Caspase-3 (sc-7148) and CUL-1 (sc-11384) were purchased from Santa Cruz Biotechnology (Santa Cruz, CA, USA).

### Survival analysis using osteosarcoma online database

Patient data was obtained from the ‘R2: Genomics Analysis and Visualization Platform (http://r2.amc.nl; http://r2platform.com, accession ID: Kuijjer - 127 - vst - ilmnhwg6v2). Survival analysis was performed using R packages “survival” and “survminer”. Median expression of Skp2 mRNA was used as a cutoff to distinguish low versus high levels of expression. Raw microarray expression data of osteosarcoma patients were retrieved from the GEO database (GSE42352).

### Immunohistochemistry (IHC)

Tumor and lung tissues were fixed in formalin, sectioned, and examined by hematoxylin-eosin (HE) staining. The Histology and Comparative Pathology Core Facility at AECOM performed Immunohistochemistry of Skp2. After deparaffinization, rehydration, and blocking, sections were incubated with an anti-Skp2 antibody (2652, Cell Signaling Technology, Beverly, MA) at 4 °C overnight, followed by incubation with a secondary antibody for 30 minutes at room temperature. The immunostaining was visualized with diaminobenzidine (Sigma, St. Louis, MO) after 5 minutes at room temperature. Slides were counter-stained with hematoxylin.

### Tissue microarrays and survival analysis

Patient OS tissue microarrays (TMA) were purchased from Novus Biologicals. After IHC using the Skp2 antibody, stained slides were graded by three independent pathologists (E.V-S, K.S, and J.S.) without previous knowledge of patient outcomes (see Acknowledgement). The staining intensity was graded as negative, weak (+), intermediate (++), and strong (+++) based on the percentage of Skp2 positive tumor cells (negative =<1% staining; (+) = 1–10% staining; (++) = 10–50% staining; (+++) =>50% staining). The percentage of stained cells in each sample was assessed in 10 random microscopic fields. Kaplan-Meier curves were generated to represent overall survival, and the log-rank test was used to compare survival probability.

### Skp2 knockdown by shRNA

SureSilencing shRNA Plasmids (336313 KH00232N) were purchased from Qiagen (Hilden, Germany). Transfection of 143B and SaOS-2 cell lines was performed with Lipofectamine 2000 (Life Technologies, Carlsbad, CA) and stable clones were selected in medium containing G418 (Gibco, Carlsbad, CA). The efficient knockdown sequences were as follows:

- Insert sequence for 143B: TCAGATCTCTCTACTTTAGTT;
- Insert sequence for SaOS-2: GGACCTATCGAACTCAGTTAT;
- Negative control: GGAATCTCATTCGATGCATAC.

Expression of Skp2 was also knocked-down in 143B and SaOS-2 cells by transient transfection of shRNA expressing plasmids followed by antibiotic selection. A HuSH shRNA control plasmid pGFP-V-RS with scrambled shRNA sequence (TR30013) and two 29-mer HuSH shRNA plasmids targeting Skp2 (SR073262 / TG301684A-D) were purchased from Origene (Rockland, MD). Cells were transiently transfected with 8 µg of the indicated plasmids using Lipofectamine 2000, incubated overnight, and selected with 2 µg/mL puromycin for 48 hours. Transfection and selection efficiency was confirmed by the presence of GFP co-expression. Selected cells were used within 48 hours of selection without further modification.

Catalog #

- Negative Control: TR30013
- Insert sequence 1: TG301684C
- Insert sequence 2: TG301684D.

### Skp2 overexpression

Overexpression of SKP2 was performed by lentivirus mediated transduction using standard protocols. Briefly, virus particles were generated using HEK293T cells (ATCC) transfected with CMV-SKP2 or CMV-GFP lentivirus transfer plasmids (a kind gift from Dr. Liang Zhu, Albert Einstein College of Medicine) and packaged with PMD2.G and pSPAX2 (Addgene). Virus-containing supernatant was collected between 24 and 72 hours post-transfection and concentrated with Lenti-X concentrator (Clonetech) according to the manufacturer’s suggested protocol. 143B and SaOS-2 cells were then transduced by addition of either GFP control or Skp2 particles in presence of 8 µg/mL polybrene, and expression of the target proteins was confirmed by fluorescence microscopy and qRT-PCR. The resulting cell lines were used for downstream assays without further modification.

### Fluorescence-activated cell sorting (FACS)

143B and SaOS-2 cells were seeded at a density of 4 × 10^5^ cells/well for 24 hours in 6-well plates. Cells were treated with 0.1% DMSO or with FKA for 24 hours. Apoptosis assays were conducted as previously described^47^. Cell cycle analysis was conducted with Click-iT^TM^ plus EdU flow cytometry assay kits (C10634) and the LIVE/DEAD (LD) Fixable dead cell stain kits (L34974, Thermo Fisher Scientific, Cambridge, MA) as previously described^50^. FlowJo^TM^ software (Tree Star Inc.) was utilized for data analysis.

### Zymogram assay

143B and SaOS cells were treated with FKA for 48 hours. Conditioned media were collected and concentrated using Pierce concentrator columns (ThermoFisher Scientific). The gelatinase activity of MMP-9 was analyzed by Novex 10% Zymogram Plus gel electrophoresis (ThermoFisher Scientific). Gels were stained with Coomassie blue and destained according to the manufacturer’s protocol. Gelatinase activity was visualized as clear bands on the gel.

### Osteosarcoma xenograft models

#### *In vivo* effects of Skp2 knockdown

Animal experiments were approved by the Institutional Animal Care Utilization Committee (IACUC) of Albert Einstein College of Medicine. 143B/shSkp2 or 143/shctrl cells were grown to 80% confluence and implanted orthotopically into the proximal tibia of SCID mice at 5 × 10^5^ cells (N = 8 animals in each group). Amputation of tumor-bearing legs of all mice was performed when any tumor reached 1.5 cm in diameter. Tumor tissues were harvested, measured, weighed and fixed in 10% formalin. Mice were then raised for two additional weeks after amputation and euthanized according to an IACUC-approved protocol. The lungs were harvested, weighed, and fixed in Bouin’s solution (Sigma-Aldrich Co., St. Louis, MO) to visualize and count surface nodules.

#### *In vivo* effects of FKA

1 × 10^6^ 143B cells were injected into SCID mice through the tail vein. Animals were randomized into 2 groups (n = 8 per group): 0.1% DMSO or FKA (200 mg/kg per day). Treatment was initiated three days after injection of tumor cells. After 28 days, the animals were sacrificed according to an IACUC-approved protocol. The lungs were harvested, weighed, and fixed in Bouin’s solution.

### Tumor Volume

Tumor size was measured once weekly by a single trained observer using an electronic caliper, starting on Day 7 after implantation. To assess tumor volume, the greatest longitudinal diameter (length) and the greatest transverse diameter (width) were measured. Tumor volumes were calculated as previously described^49^.

### Statistical analysis

Patient demographic data from each cohort was reported as frequencies for categorical variables and means with standard deviations for continuous variables. The difference between two groups was assessed using an unpaired 2-tailed Student t-test. Differences in cell proliferation and viability were analyzed by two-way ANOVA. Statistical significance was set at p < 0.05.

## Electronic supplementary material

Supplemental Figures 1–11 & Original Western Blots
