# Supplementary material for: Down-regulation of Skp2 expression inhibits invasion and lung metastasis in osteosarcoma
Source: Sci Rep. 2018 Sep 24;8:14294. doi: 10.1038/s41598-018-32428-9 (PMC6155331; doi:10.1038/s41598-018-32428-9)

Down-regulation of Skp2 expression inhibits invasion and lung metastasis in osteosarcoma

Authors and affiliations:

Yidan Zhang[1,7], Yoav S. Zvi[2], Brian Batko[2], Nikolas Zaphiros[2], Edmond O'Donnell[2], Jichuan Wang[2,7], Kenji Sato[2], Rui Yang[2], David S. Geller[2], Pratistha Koirala[1], Wendong Zhang[1], Xiuquan Du[2], Sajida Piperdi[1], Yang Liu[3], Deyou Zheng[3,4], Michael Roth[5], Jonathan Gill[5], Jinghang Zhang[6], Tingting Ren[7], Richard Gorlick[5], Xiaolin Zi[8], Bang H. Hoang*[2]

[1]Division of Pediatric Hematology, Oncology, Marrow & Blood Cell Transplantation, Children's Hospital at Montefiore, Albert Einstein College of Medicine, Bronx, NY

[2]Department of Orthopedic Surgery, Montefiore Medical Center, Albert Einstein College of Medicine, Bronx, NY

[3]Department of Genetics, Albert Einstein College of Medicine, Bronx, NY

[4]Department of Neurology, Department of Genetics and Neuroscience, Albert Einstein College of Medicine, Bronx, NY

[5]Division of Pediatrics, University of Texas MD Anderson Cancer Center, Houston, TX

[6]Flow Cytometry Core, Albert Einstein College of Medicine, Bronx, NY

[7]Musculoskleletal Tumor Center, Beijing Key Laboratory for Musculoskeletal Tumors, Peking University People's Hospital, Beijing, China

[8]Department of Urology, University of California, Irvine Medical Center, Orange, CA

Keywords (5): S-phase kinase-associated protein 2, Osteosarcoma, Flavokawain A, Lung metastasis

Additional information:

Financial support: Orthopedic Research and Education Foundation, Montefiore Medical Center, and Sarcoma Strong (to B. H. Hoang) and NIH award R01CA193967 (to X. Zi.).

Corresponding author: Bang H. Hoang

Mailing address: 3400 Bainbridge Ave, 6th Floor, Bronx, NY, 10476

Phone number: (718) 920-2060

Fax number: (718) 653-1587

Email address: bahoang@montefiore.org

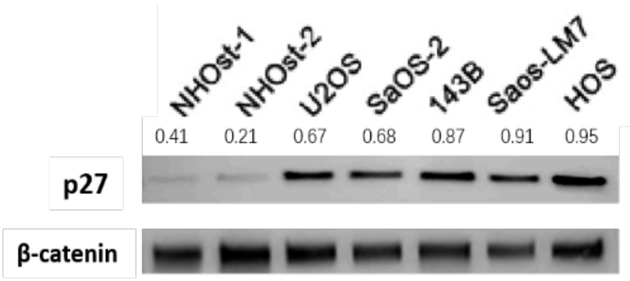

Supplement Figure 1. Immunoblotting of p27. Compared to NHOsts, p27 protein levels are overexpressed in standard osteosarcoma cell lines.

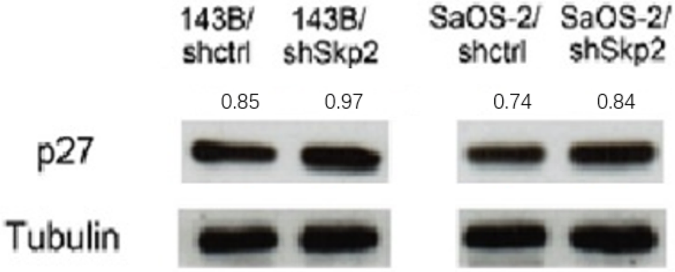

Supplement Figure 2. Immunoblotting data under the same experiment condition in Fig. 2. No significant correlation between Skp2 knockdown and p27 protein levels was observed in 143B and SaOS-2 cell lines.

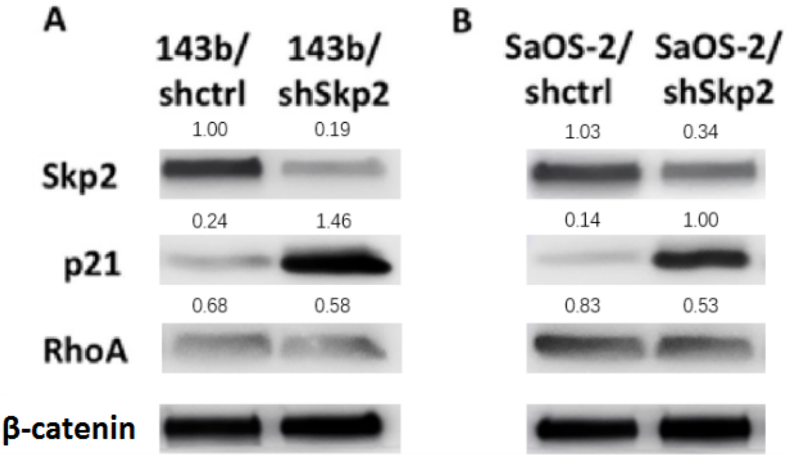

Supplement figure 3. Effects of genetic knockdown of Skp2 using additional shRNA construct. (A-B) Osteosarcoma cell lines 143b and SaOS-2 were transfected with an additional unique shRNA construct targeting Skp2. Similar effects on Skp2 targets (p21 and RhoA) were observed across three different shRNA constructs, confirming the specificity of Skp2 knockdown results.

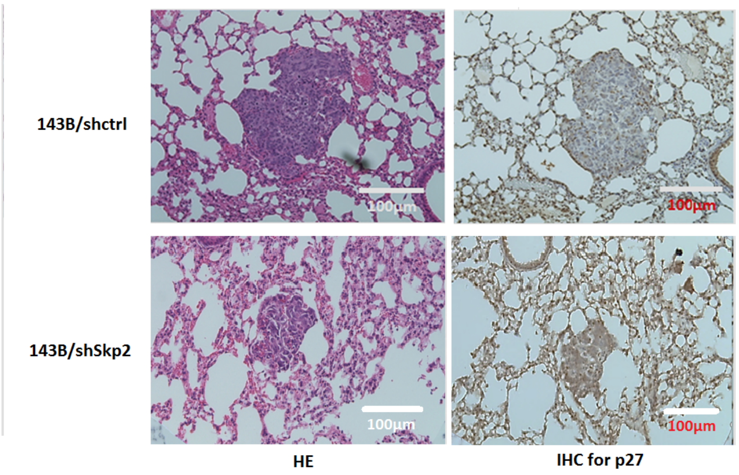

Supplement Figure 4. Representative pictures of hematoxylin-eosin (HE) and IHC staining of lung tissues using the same experimental condition in Fig. 3H. No significant correlation between Skp2 knockdown and p27 protein levels was observed by immunostaining in metastatic lung nodules.

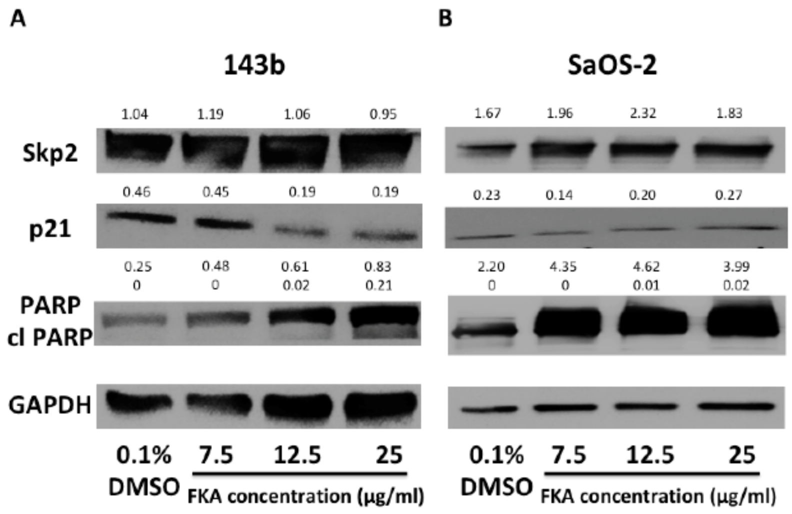

Supplement figure 5. Overexpression of Skp2 abrogates the effects of FKA in osteosarcoma cell lines. **(A-B)** Skp2 was overexpressed in osteosarcoma cell lines 143B and SaOS-2 using a lentiviral Skp2 construct. Treatment with FKA for 48 hours showed that Skp2 overexpression abrogated the effects of FKA on p21 and cleaved PARP.

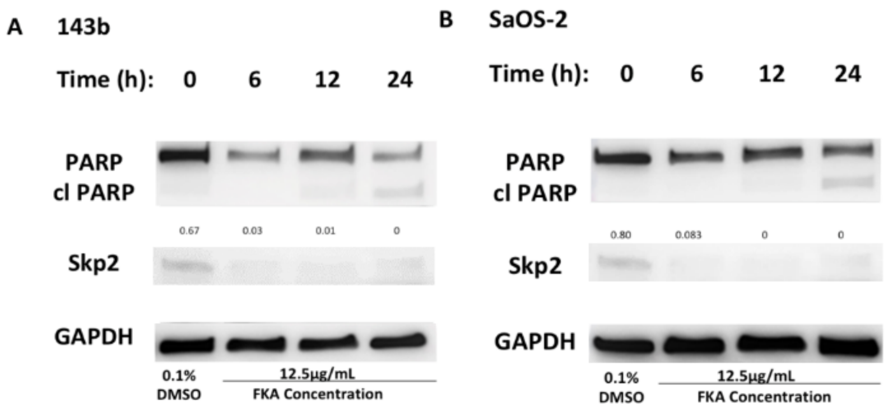

Supplement figure 6. FKA-mediated suppression of Skp2 precedes cellular apoptosis. **(A-B)** Osteosarcoma cell lines (143b and SaOS-2) were treated with vehicle control or FKA (12.5μg/mL) for indicated time periods (6, 12, and 24 hours) and harvested for western blots. Suppression of Skp2 by FKA was observed at 6 hours, while apoptosis by PARP cleavage was seen at later time periods (12-24 hours).

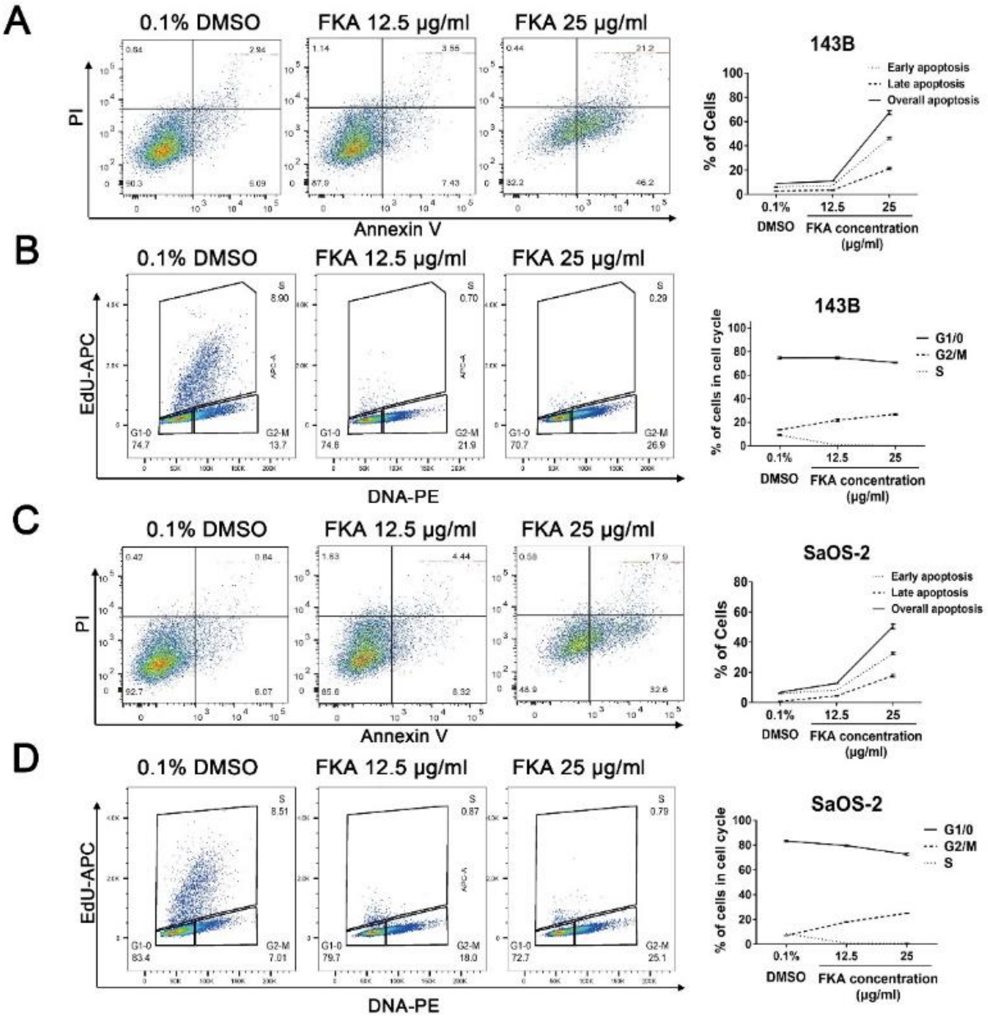

Supplement figure 7. FKA treatment induces apoptosis and G2/M arrest in osteosarcoma cell lines. **(A and B)** FACS analysis in 143B cells following 24 hours of exposure to FKA. Apoptosis was seen in 143B cells at a higher concentration of FKA (25 μg/ml) **(A)**. Cell cycle arrest in G2/M phase was observed after FKA treatment **(B)**. **(C and D)** FACS analysis in SaOS-2 cells following 24 hours of exposure to FKA. Apoptosis was also detected in SaOS-2 cells at a high concentration of FKA (25 μg/ml) **(C)**. Similar to 143B, an arrest in G2/M was also demonstrated for SaOS-2 **(D)**.

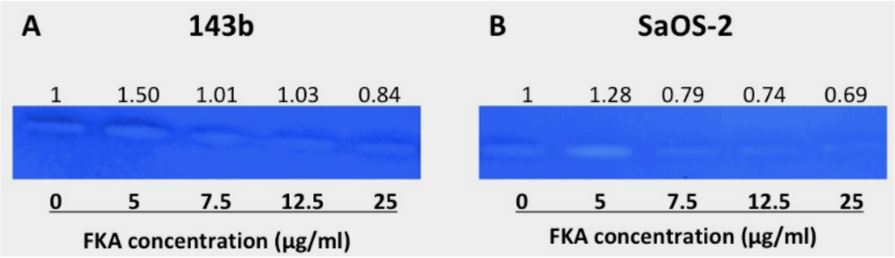

Supplement Figure 8. Effects of FKA treatment on the gelatinase activity of MMP-9. **(A-B)** Osteosarcoma cell lines 143b and SaOS-2 were treated with FKA for 48 hours. Zymogram assays as described in Materials and Methods were performed to determine the gelatinolytic activity of MMP-9.

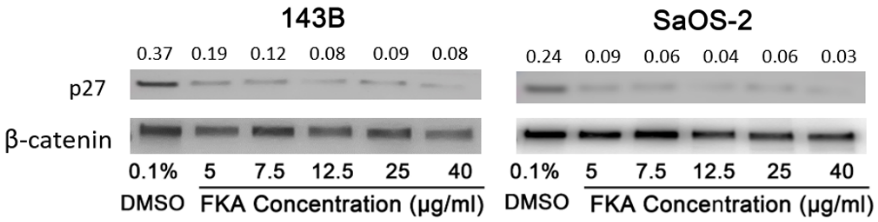

Supplement Figure 9. Immunoblotting of p27 under the same experiment condition in Fig. 5C. Treatment with FKA markedly decreased Skp2 and p27 protein levels in 143B and SaOS-2 cell lines.

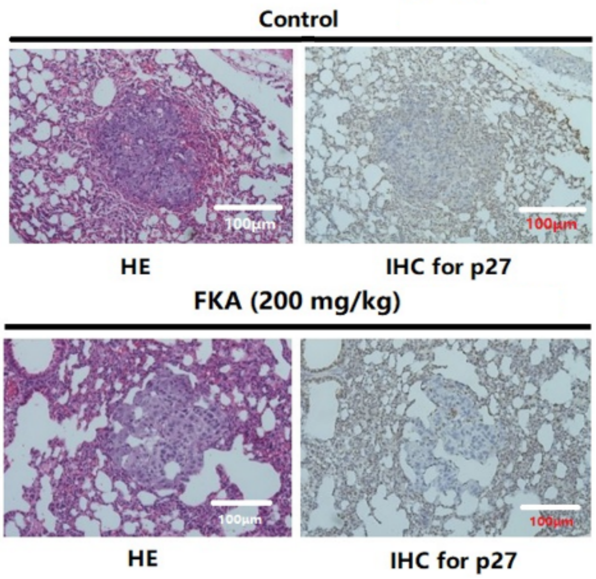

Supplement Figure 10. Representative pictures of hematoxylin-eosin (HE) and IHC staining of lung tissues under the same experimental condition as Fig. 6F. Lower levels of p27 immunostaining were detected in metastatic lung nodules from the FKA-treated group compared to control.

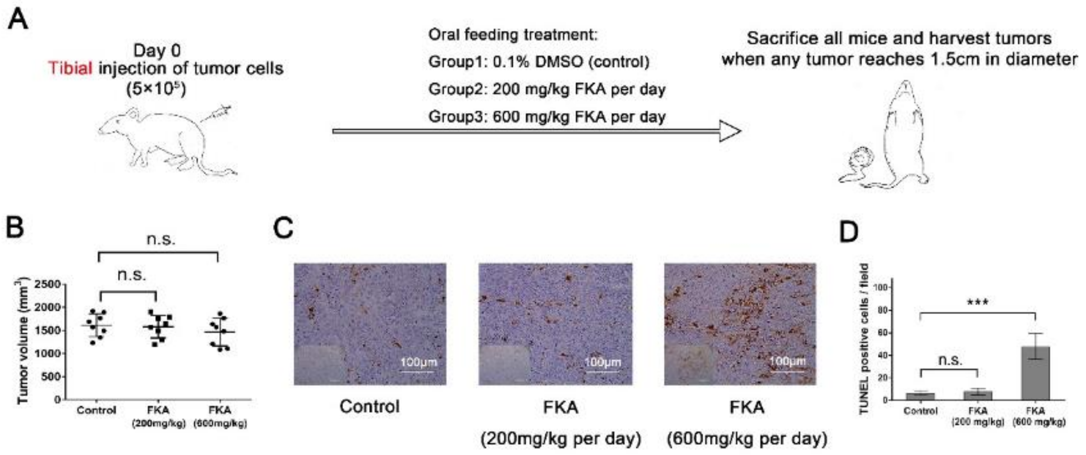

Supplement figure 11. *In vivo* efficacy of FKA in an orthotopic osteosarcoma model. **(A)** $5×10^5$ 143B cells were injected into the proximal tibia of SCID mice. Three days after injection, animals were treated daily with 200 mg/kg, 600mg/kg of oral FKA, or vehicle control. Hind limbs of all animals were harvested once any tumor reached 1.5 cm in diameter. **(B)** Tumor size. There was no significant difference in tibial tumor size between FKA-treated groups and the control group. **(C)** Representative images of TUNEL assays in tumor tissues. **(D)** Compared to the control group, a significantly greater number of apoptotic cells were detected in tumors treated with 600mg/kg/day of FKA. No significant difference in apoptosis was seen between vehicle control and 200 mg/kg FKA-treated groups. Independent t-tests (two-tailed) were used to analyze the difference between vehicle control and each of the FKA-treated groups. Statistical significance is indicated by: N.S. = no significant difference; (***) $p < 0.001$. Column: mean; Error bars: SD.

Figures of Full Length Western Blots

1. Western blot in Figure1B

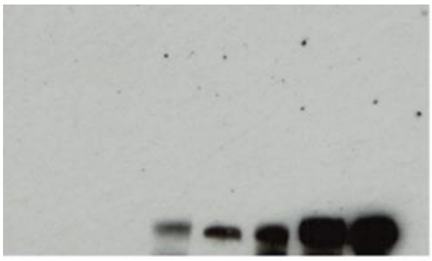

Skp2 (48,46kDa)

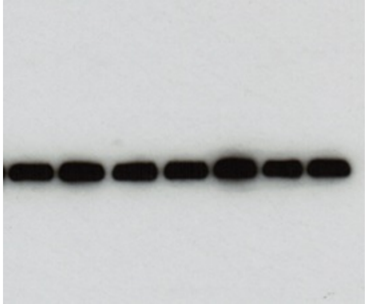

β-Tubulin (55kDa)

2. Western blot in Figure1C

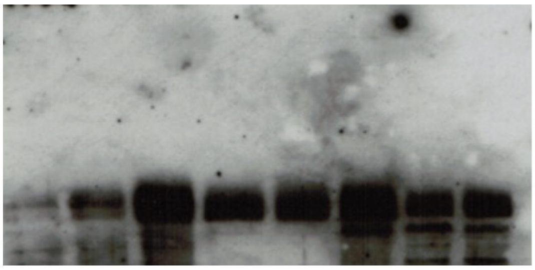

Skp2 (48, 46kDa)

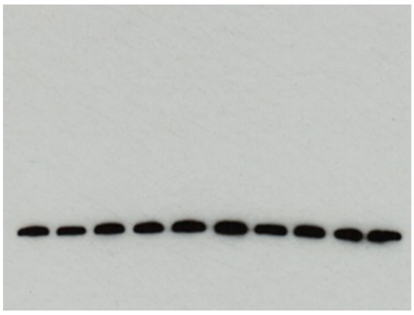

β-Tubulin (55kDa)

3. Western blot in Figure2I and 6. Supplementary Figure 2

p27 (27kd)

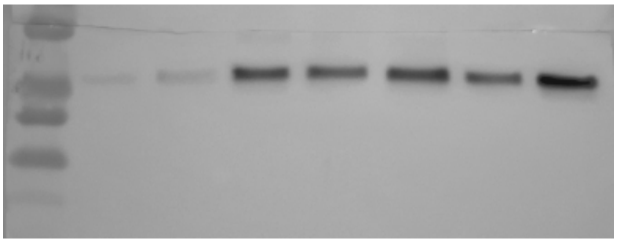

β-catenin(92kd)

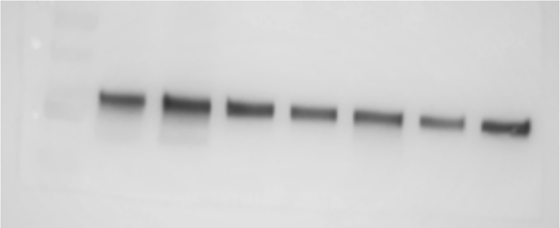

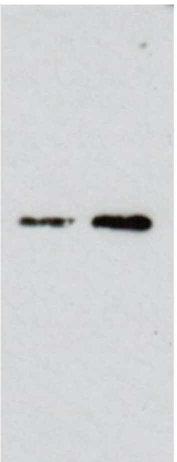

p21 (21kDa)

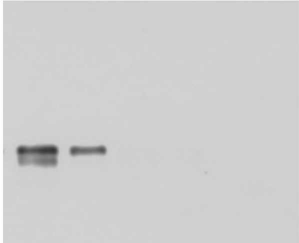

Skp2 (48,46kDa)

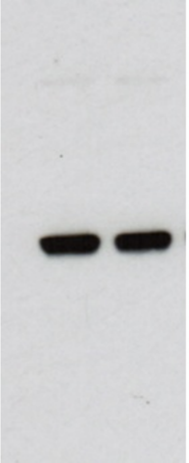

Caspase-3 (35kDa)

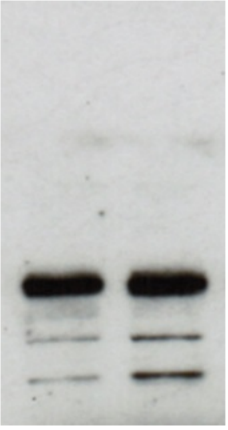

cl Caspase-3 (17kDa)

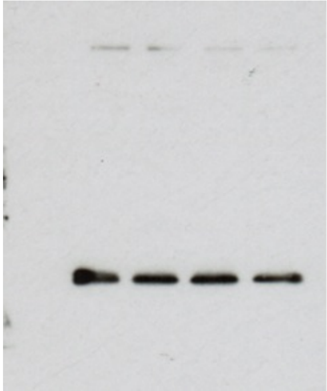

RhoA (21kDa)

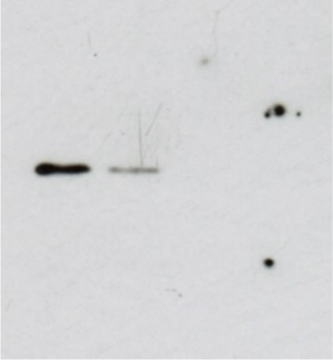

MMP-9 (92kDa)

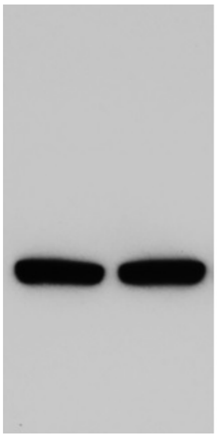

β-Tubulin (55kDa)

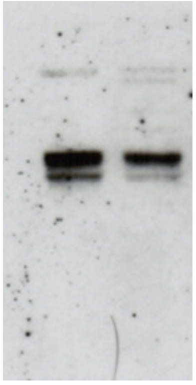

Skp2 (48,46kDa)

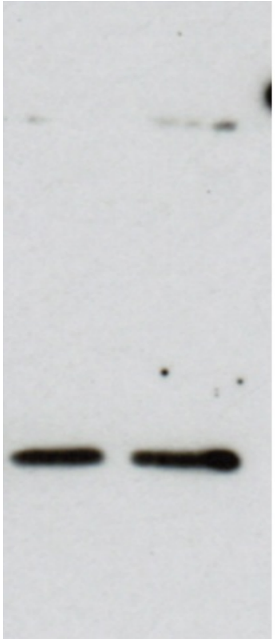

p21 (21kDa)

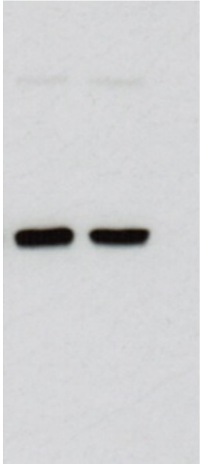

Caspase-3 (35kDa)

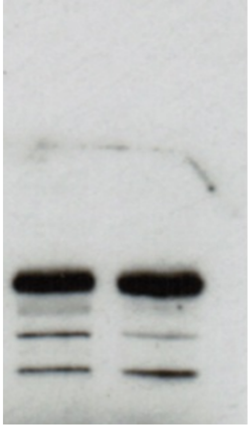

cl Caspase-3 (17kDa)

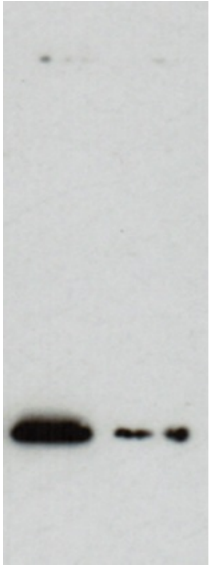

RhoA (21kDa)

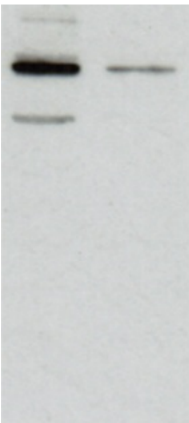

MMP-9 (92kDa)

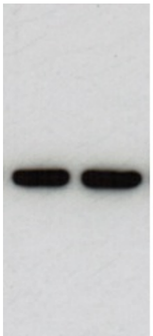

β-Tubulin (55kDa)

4. Western blot in Figure3G

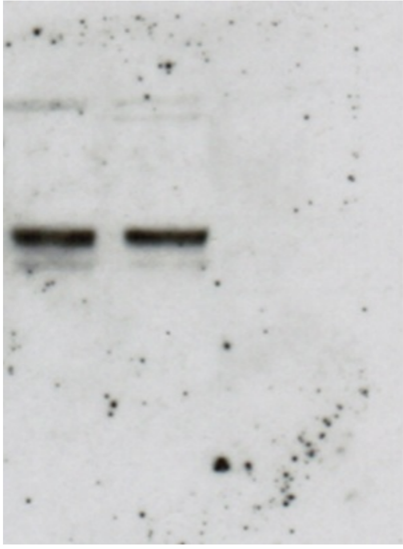

Skp2 (48,46kDa)

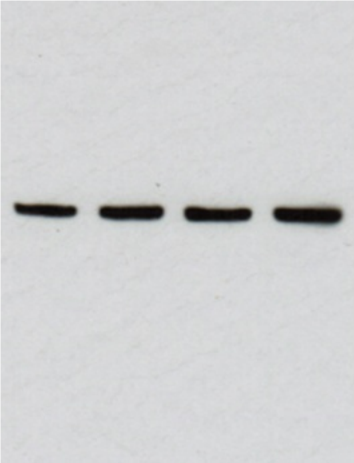

β-Tubulin (55kDa)

5. Western blot in Figure5C

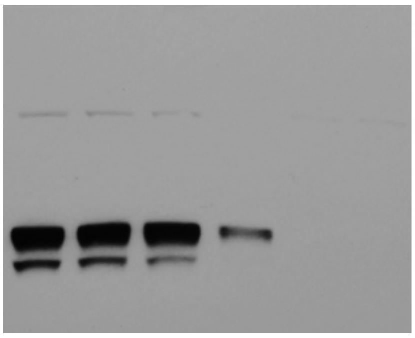

Skp2 (48,46kDa)

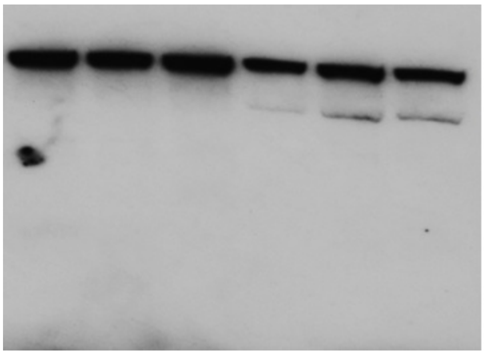

Cul-1 Nedd8
Cul-1 (90kDa)

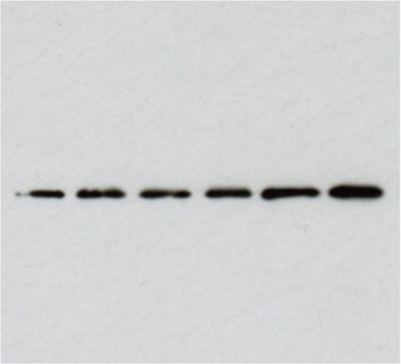

p21 (21kDa)

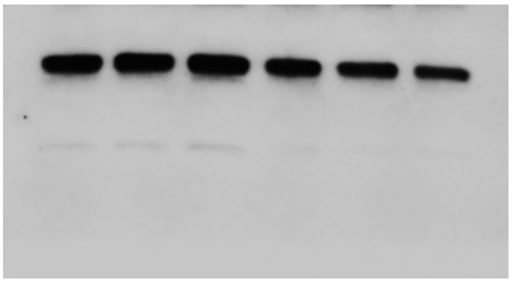

Caspase-3 (35kDa)

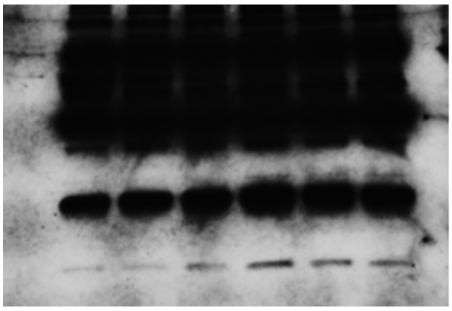

cl Caspase-3 (17kDa)

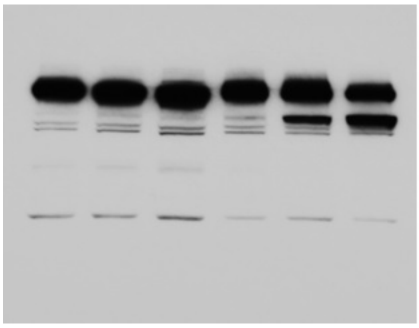

PARP (116 kDa)
Cl PARP (89 kDa)

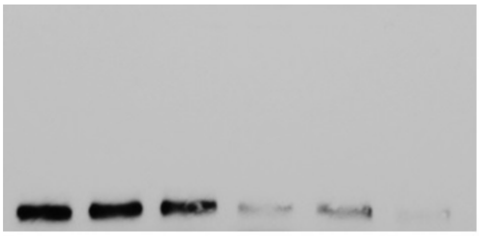

RhoA (21kDa)

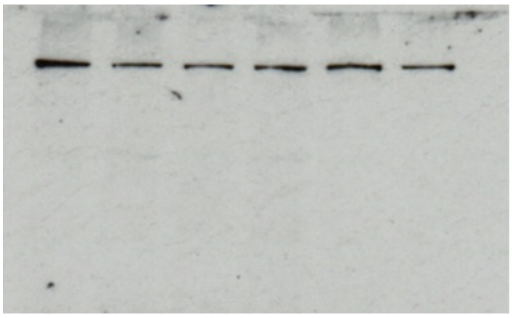

MMP-9 (92kDa)

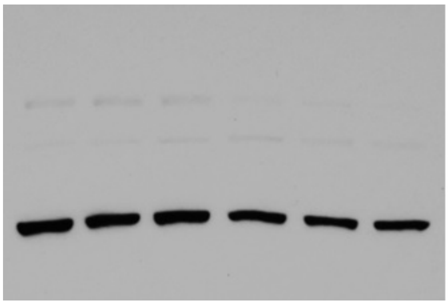

β-Tubulin (55kDa)

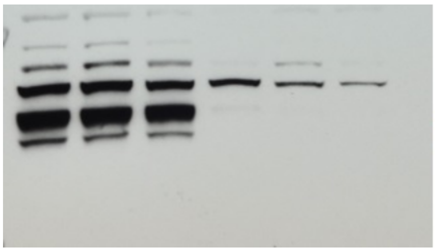

Skp2 (48,46kDa)

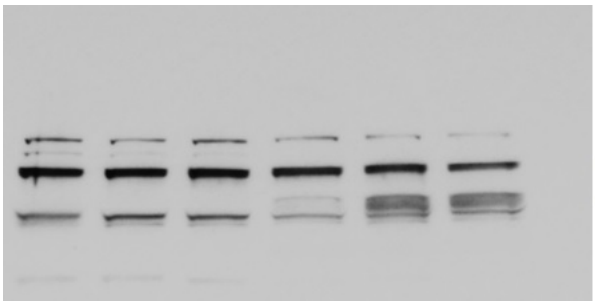

Cul-1 Nedd8
Cul-1 (90kDa)

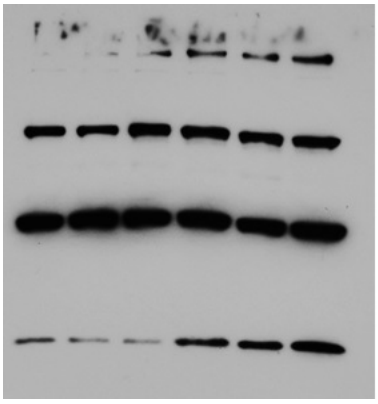

p21 (21kDa)

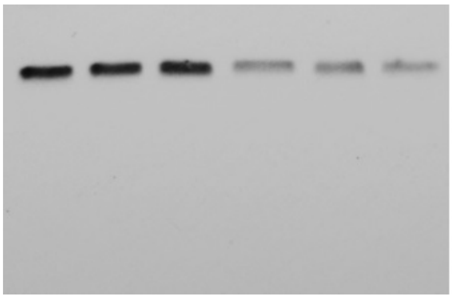

Caspase-3 (35kDa)

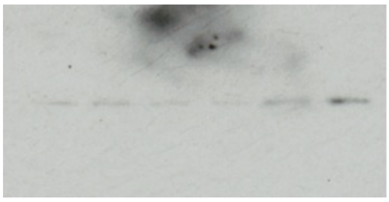

cl Caspase-3 (17kDa)

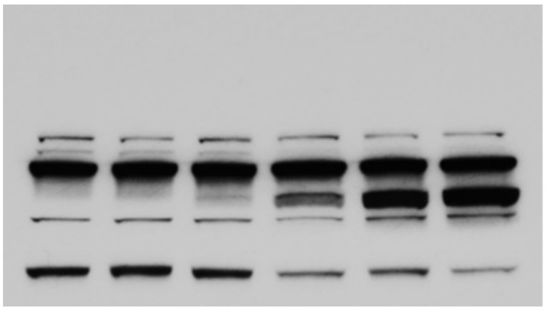

PARP (116 kDa)
Cl PARP (89 kDa)

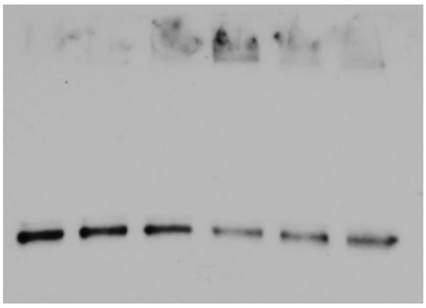

RhoA (21kDa)

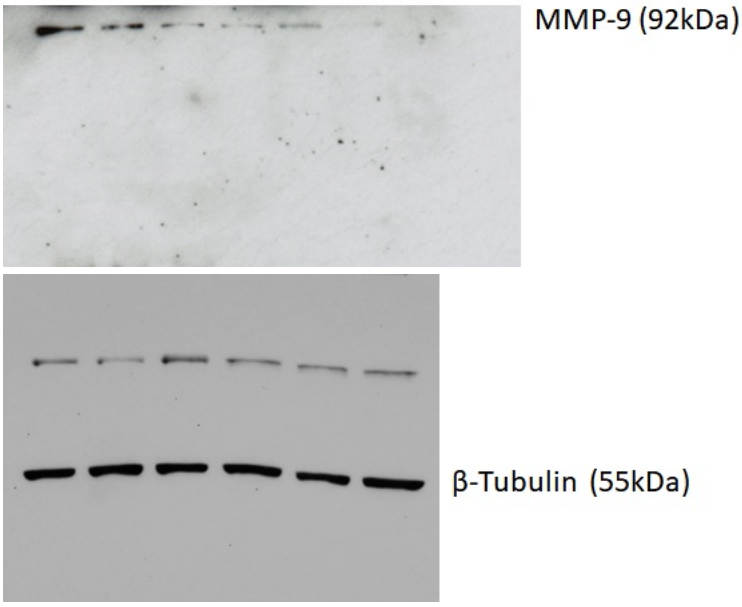

6. Western blot in Supplementary Figure 1

p27 (27kd)

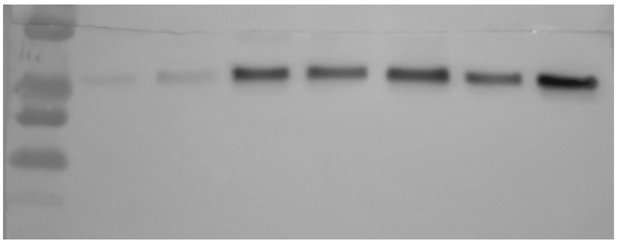

β-catenin(92kd)

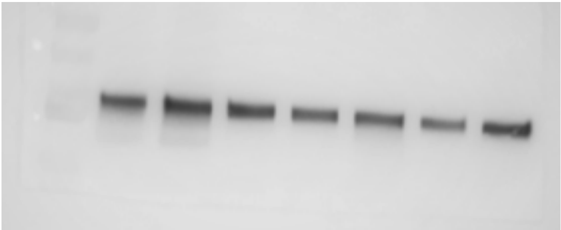

7. Western blot in Supplementary Figure 3

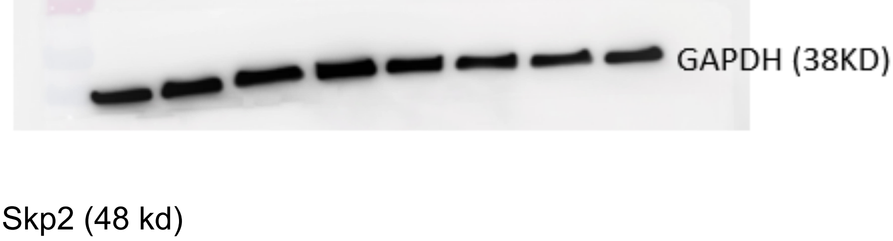

Skp2 (48 kd)

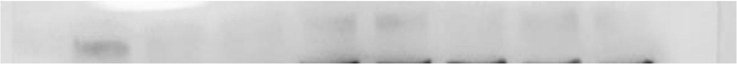

8. Western blot in Supplementary Figure 4

Skp2 (48 kd), β-catenin(92kd)

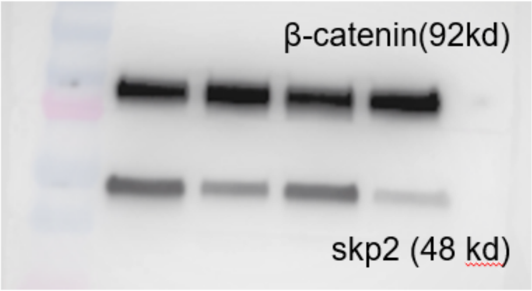

RhoA (21kd)

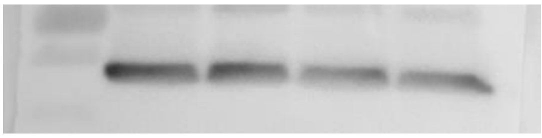

p21 (21kd)

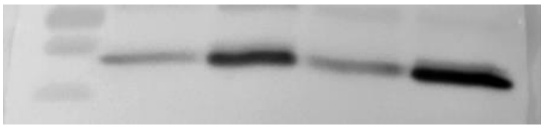

9. Western blot in Supplementary Figure 5

PARP and cl PARP (89kd)

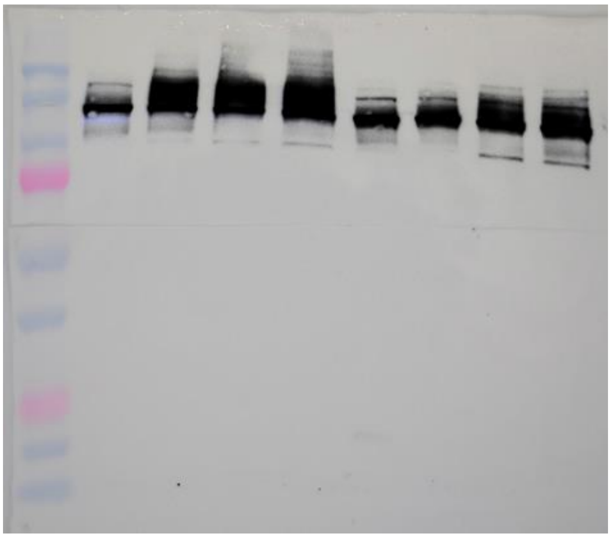

Skp2 (48kd) and GAPDH (37kd)

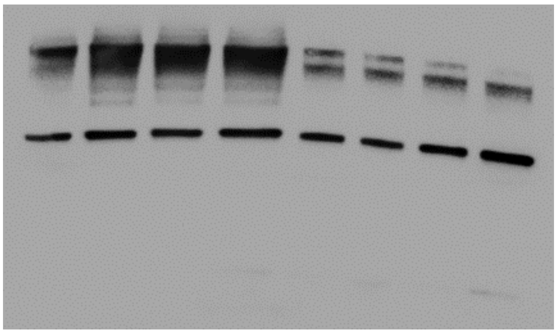

p21 (21kd)

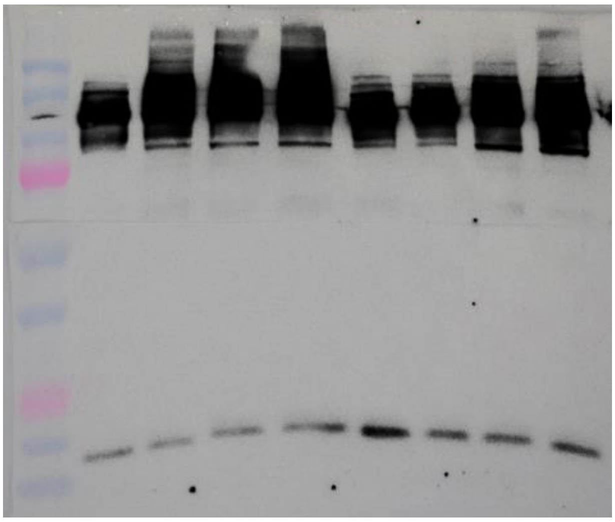

PARP and cl PARP (89kD)

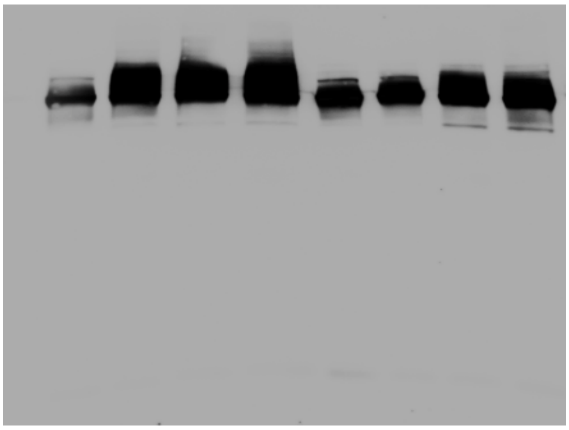

Skp2 (48kd) and GAPDH (37kd)

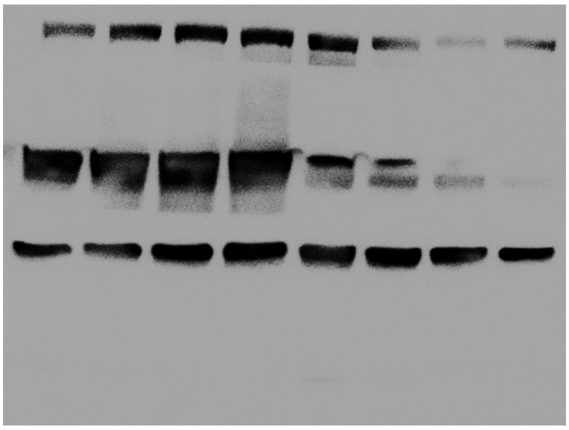

p21 (21kd)

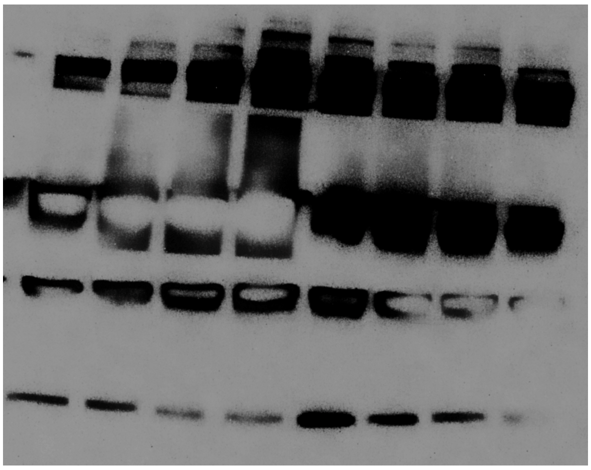

10. Western blot in Supplementary Figure 10

β-catenin(92kd) In SaoS-2 cell line

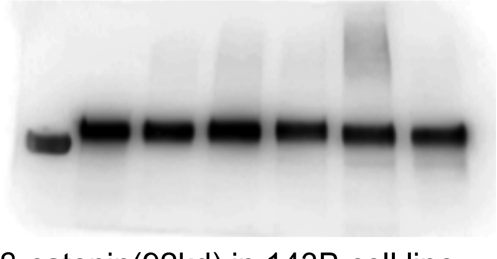

β-catenin(92kd) in 143B cell line

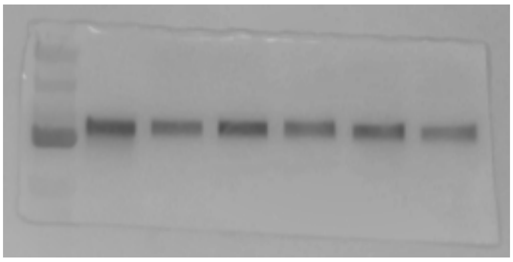

p27 (27kd) in SaoS-2 cell line

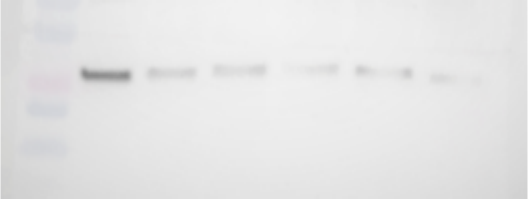

p27 (27kd) in 143B cell line

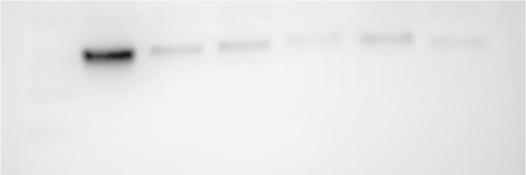

Supplement: Supplementary file 1 — Supplemental Figures 1–11 & Original Western Blots [file 41598_2018_32428_MOESM1_ESM.pdf]
